# Supplementary material for: TRIB3-EGFR interaction promotes lung cancer progression and defines a therapeutic target
Source: Nat Commun. 2020 Jul 21;11:3660. doi: 10.1038/s41467-020-17385-0 (PMC7374170; doi:10.1038/s41467-020-17385-0)
Supplement: Supplementary file 1 — Supplementary Information [file 41467_2020_17385_MOESM1_ESM.pdf]

TRIB3-EGFR interaction promotes lung cancer progression and defines a therapeutic target

Yu et al.

Supplementary Figures and Legends

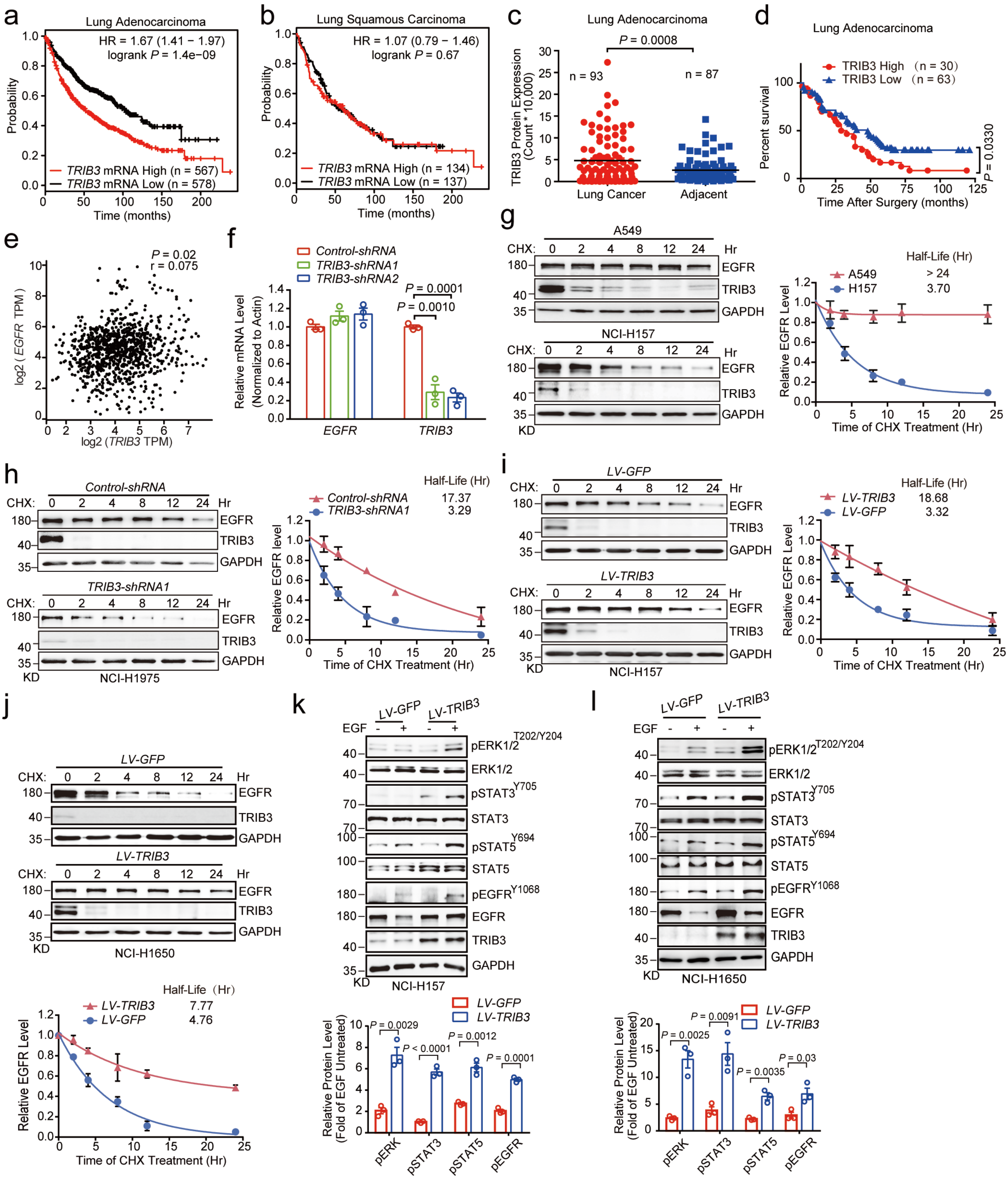

Supplementary Figure 1 TRIB3 enhances EGFR stability and its signaling activity.

a,b Kaplan–Meier survival curves relative to *TRIB3* mRNA level were generated for lung adenocarcinoma (a) and lung squamous carcinoma (b) via online Kaplan-Meier Plotter (two-sided). **c,** Quantitative analysis of TRIB3 protein expression in paired lung adenocarcinoma clinical samples. Statistical significance was determined by two-tailed Student's t-test. **d,** Kaplan–Meier survival curves relative to TRIB3 protein level were generated for lung adenocarcinoma. Statistical difference was determined by two-sided log-rank test. **e,** Correlation between *EGFR* and *TRIB3* mRNA expression across TCGA lung cancer data sets. Analysis was performed on the following website: http://gepia.cancer-pku.cn. Pearson's correlation coefficient analysis was used to define correlations. **f,** *EGFR* and *TRIB3* mRNA expression in control or *TRIB3*-silenced A549 cells were determined by quantitative PCR (qPCR). Data represent means ± SEM of 3 independent assays. **g-j,** Protein stability of EGFR was determined by IB in different NSCLC cell lines with TRIB3 manipulation. A549 and NCI-H157 cells (g); NCI-H1975 cells stably expressed *control-shRNA* or *TRIB3-shRNA1* plasmid (h); NCI-H157 cells with or without *TRIB3* stably ectopic expression (i); and NCI-H1650 cells with or without *TRIB3* stably ectopic expression (j). Cells were treated with CHX (10 μg ml^{-1}) at indicated intervals and analyzed by IB analysis. Data are means ± SEM of 3 independent assays. **k,l,** EGFR and its signaling proteins in: NCI-H157 cells with or without *TRIB3* stably ectopic expression (k); and NCI-H1650 cells with or without *TRIB3* stably ectopic expression (l). Cells were stimulated with or without EGF (100 ng ml^{-1}) for 1 hr and analyzed by IB analysis. Data represent means ± SEM of 3 independent assays. Statistical significance between

two groups was determined with two-tailed Student's t-test. Source data are provided as a Source Data file. (Lentiviral: LV)

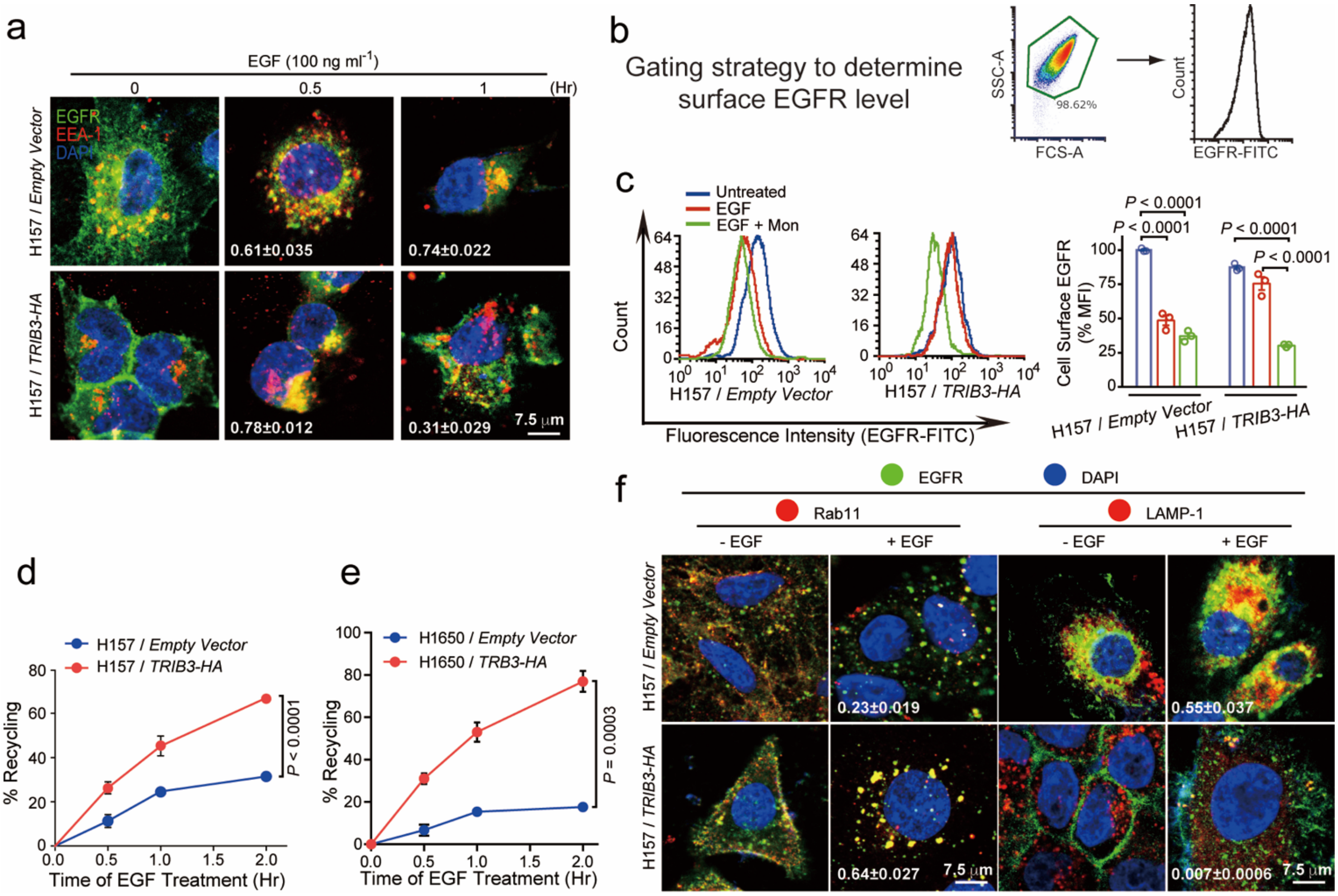

a
EGF (100 ng ml-1)
0
0.5
1
(Hr)
EGFR
EEA-1
DAPI
H157 / Empty Vector
H157 / TRIB3-HA
0.61±0.035
0.74±0.022
0.78±0.012
0.31±0.029
7.5 μm
b
Gating strategy to determine surface EGFR level
SSC-A
FCS-A
98.62%
Count
EGFR-FITC
c
Untreated
EGF
EGF + Mon
Count
H157 / Empty Vector
H157 / TRIB3-HA
Fluorescence Intensity (EGFR-FITC)
Cell Surface EGFR (% MFI)
P < 0.0001
P < 0.0001
P < 0.0001
P < 0.0001
d
H157 / Empty Vector
H157 / TRIB3-HA
% Recycling
Time of EGF Treatment (Hr)
P < 0.0001
e
H1650 / Empty Vector
H1650 / TRB3-HA
% Recycling
Time of EGF Treatment (Hr)
P = 0.0003
f
EGFR
DAPI
Rab11
LAMP-1
- EGF
+ EGF
- EGF
+ EGF
H157 / Empty Vector
H157 / TRIB3-HA
0.23±0.019
0.55±0.037
0.64±0.027
0.007±0.0006
7.5 μm
7.5 μm

Supplementary Figure 2 Overexpression of TRIB3 promotes EGFR recycling.

a, NCI-H157 cells and NCI-H157 cells stably expressed *TRIB3* were non-stimulated or EGF (100 ng ml^{-1}) stimulated for indicated times. Co-localization of EGFR with EEA1 was analyzed with confocal microscopy, quantification of EGFR and EEA1 colocalization was shown as Pearson's coefficient. Data are means ± SEM of 3 independent assays. **b,** Gating strategy to determine surface EGFR level presented in figure 2d and supplementary figure 2c. **c,** Quantitative analyses of cell surface EGFR in NCI-H157 cells stably expressed *Empty-Vector* or *TRIB3-HA* expression plasmid. Cells were pre-incubated with DMSO or 10 μM monensin for 4 hr and treated with or without EGF (100 ng ml^{-1}) for another 1 hr. The MFI of EGFR on cell surface was detected by flow cytometry analysis. Left: representative flow cytometry data. Right: The data was normalized to H157/ *Empty-Vector* cells without EGF stimulation, which was considered as 100% MFI signal. Data are means ± SEM, n = 3. **d,e,** EGFR recycling was detected in NCI-H157 cells with or without *TRIB3-HA* stably ectopic expression (d); or in NCI-H1650 cells with or without *TRIB3-HA* stably ectopic expression (e). Data are means ± SEM of 3 independent assays. **f,** NCI-H157 cells and NCI-H157 cells stably expressed *TRIB3* were stimulated with or without EGF (100 ng ml^{-1}) for 30 min. Confocal images show the co-localization of EGFR with Rab11 and LAMP1. Quantification of EGFR/Rab11 or EGFR/LAMP-1 colocalization was shown as Pearson's coefficient. Data are means ± SEM of 3 independent assays. Statistical significance between two groups was determined with two-tailed Student's t-test. Statistical significance among groups was determined by one-way ANOVA test. Source data are provided as a Source Data file.

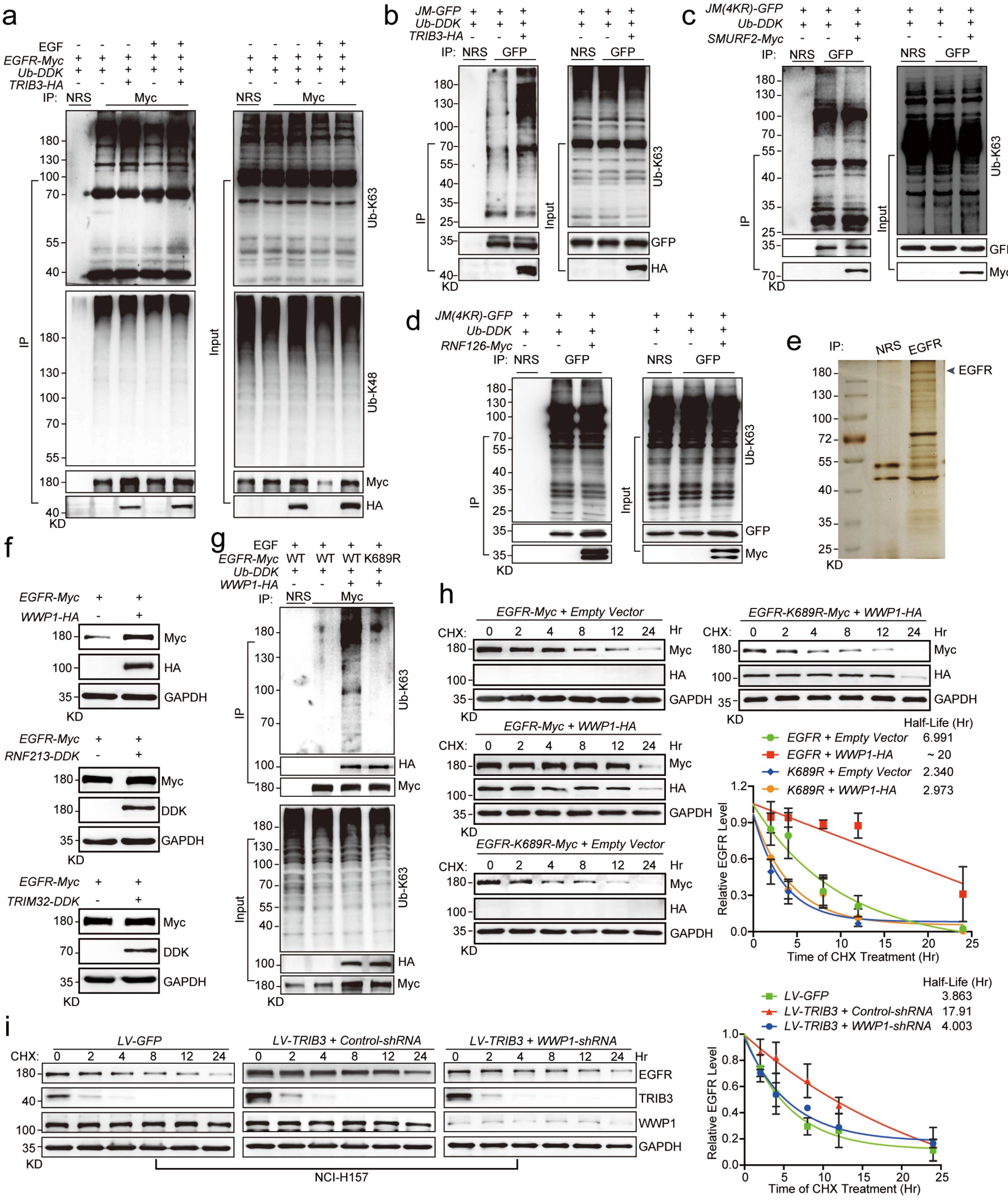
a
EGF
EGFR-Myc
Ub-DDK
TRIB3-HA
IP: NRS Myc
IP
Input
Ub-K63
Ub-K48
Myc
HA
KD
b
JM-GFP
Ub-DDK
TRIB3-HA
IP: NRS GFP
GFP
HA
c
JM(4KR)-GFP
Ub-DDK
SMURF2-Myc
d
JM(4KR)-GFP
Ub-DDK
RNF126-Myc
e
IP: NRS EGFR
EGFR
f
EGFR-Myc
WWP1-HA
RNF213-DDK
TRIM32-DDK
DDK
GAPDH
g
EGF
EGFR-Myc WT WT WT K689R
Ub-DDK
WWP1-HA
h
EGFR-Myc + Empty Vector
EGFR-K689R-Myc + WWP1-HA
EGFR-Myc + WWP1-HA
EGFR-K689R-Myc + Empty Vector
CHX: 0 2 4 8 12 24 Hr
Half-Life (Hr)
EGFR + Empty Vector 6.991
EGFR + WWP1-HA ~ 20
K689R + Empty Vector 2.340
K689R + WWP1-HA 2.973
Relative EGFR Level
Time of CHX Treatment (Hr)
i
LV-GFP
LV-TRIB3 + Control-shRNA
LV-TRIB3 + WWP1-shRNA
EGFR
TRIB3
WWP1
GAPDH
NCI-H157
Half-Life (Hr)
LV-GFP 3.863
LV-TRIB3 + Control-shRNA 17.91
LV-TRIB3 + WWP1-shRNA 4.003
Relative EGFR Level
Time of CHX Treatment (Hr)

Supplementary Figure 3 TRIB3 promotes K63-linked ubiquitination of EGFR at K689 via WWP1.

a, HEK 293T cells were transfected with indicated plasmids. 24 hr after transfection, cells were treated with or without EGF (100 ng ml^{-1}) for 30 min and cell extracts were IP with normal rabbit IgG or anti-Myc Ab. The K63-linked or the K48-linked ubiquitination of EGFR were detected by IB. **b,** HEK 293T cells were transfected with indicated plasmids. 24 hr after transfection, cell extracts were IP with normal rabbit IgG or anti-GFP Ab. The K63-linked ubiquitination of EGFR JM region was detected by IB. **c,d,** HEK 293T cells were transfected with indicated plasmids. 24 hr after transfection, cell extracts were IP with normal rabbit IgG or anti-GFP Ab. The K63-linked ubiquitination of EGFR JM region was detected by IB. **e,** Total cell lysate extract from A549 cells was subjected to affinity purification. The purified protein complex was resolved on SDS-PAGE and silver stained, then the bands were retrieved and analyzed by MS analysis. **f,** HEK 293T cells were transfected with indicated plasmids. 24 hr after transfection, expression of EGFR-Myc was detected by IB. **g,** HEK 293T cells were transfected with indicated plasmids. The WWP1-induced K63-linked ubiquitination of EGFR wild-type and the K689R mutant was analyzed by IB. **h,** HEK 293T cells transfected with indicated plasmids were treated with CHX (10 μg ml^{-1}) at indicated intervals, then cell lysates were analyzed by IB. Data are means ± SEM, n = 3. **i,** NCI-H157 cells stably expressed with indicated plasmids were treated with CHX (10 μg ml^{-1}) at indicated intervals. Cell lysates were analyzed by IB. Data are means ± SEM, n = 3. Source data are provided as a Source Data file.

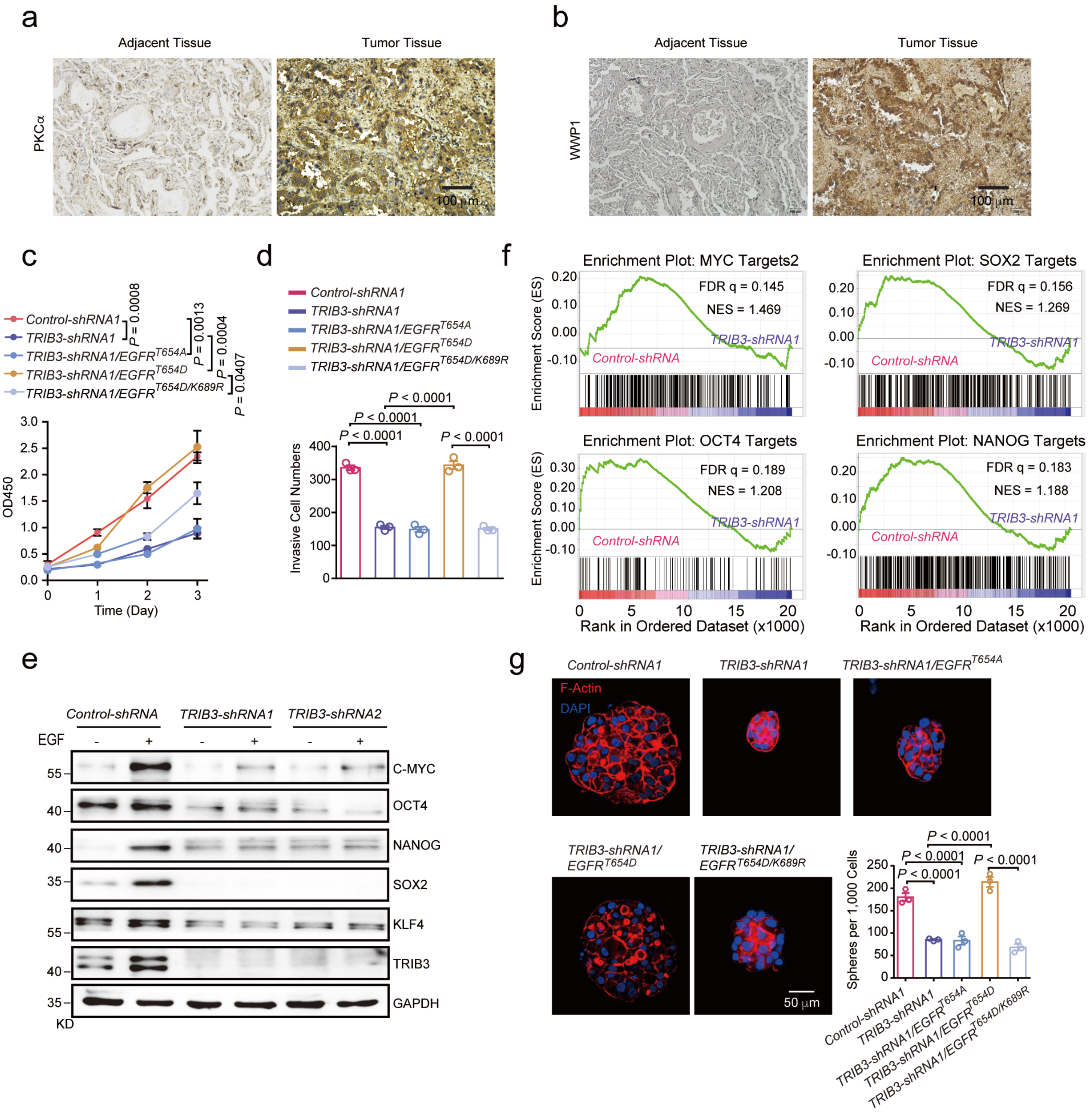
a
Adjacent Tissue
Tumor Tissue
PKCα
100 μm
b
Adjacent Tissue
Tumor Tissue
WWP1
100 μm
c
Control-shRNA1
TRIB3-shRNA1
TRIB3-shRNA1/EGFR T654A
TRIB3-shRNA1/EGFR T654D
TRIB3-shRNA1/EGFR T654D/K689R
P = 0.0008
P = 0.0013
P = 0.0004
P = 0.0407
OD450
Time (Day)
d
Control-shRNA1
TRIB3-shRNA1
TRIB3-shRNA1/EGFR T654A
TRIB3-shRNA1/EGFR T654D
TRIB3-shRNA1/EGFR T654D/K689R
P < 0.0001
Invasive Cell Numbers
f
Enrichment Plot: MYC Targets2
FDR q = 0.145
NES = 1.469
Enrichment Plot: SOX2 Targets
FDR q = 0.156
NES = 1.269
Enrichment Plot: OCT4 Targets
FDR q = 0.189
NES = 1.208
Enrichment Plot: NANOG Targets
FDR q = 0.183
NES = 1.188
Control-shRNA
TRIB3-shRNA1
Enrichment Score (ES)
Rank in Ordered Dataset (x1000)
e
Control-shRNA
TRIB3-shRNA1
TRIB3-shRNA2
EGF
C-MYC
OCT4
NANOG
SOX2
KLF4
TRIB3
GAPDH
KD
g
Control-shRNA1
TRIB3-shRNA1
TRIB3-shRNA1/EGFR T654A
F-Actin
DAPI
TRIB3-shRNA1/EGFR T654D
TRIB3-shRNA1/EGFR T654D/K689R
50 μm
P < 0.0001
Spheres per 1,000 Cells

Supplementary Figure 4 TRIB3 promotes lung cancer stemness by promoting EGFR recycling.

a,b Representative immune-histological staining of PKCα (a, n = 91 of lung cancer tissues and n = 87 of adjacent tissues) and WWP1 (b, n = 92 of lung cancer tissues and n = 87 adjacent tissues) in tumor and adjacent non-tumor tissues of human lung adenocarcinoma. **c,** Cell proliferation was measured by CCK-8 assay in A549 cells stably transfected with indicated plasmids. Data are means ± SEM, n = 4. **d,** The invasive capacities of A549 cells stably transfected with indicated plasmids were evaluated with transwell assays. Data are means ± SEM, n = 3. **e,** A549/*Control-shRNA*, A549/*TRIB3-shRNA1* and A549/*TRIB3-shRNA2* cells were treated with EGF or not. The stem cell markers were detected by IB. Data are representatives of 3 independent assays. **f,** GSEA enrichment plot for the "Myc Targets2", "SOX2 Targets", "OCT4 Targets" and "NANOG Targets" gene modules in A549 cells stably expressed *control-shRNA* or *TRIB3-shRNA1*. FDR q, false discovery rate q value; NES, normalized enrichment score. **g,** Immunostaining for F-Actin and DAPI in the tumor sphere of A549 cells with indicated plasmids stably expressed. Data represent means ± SEM, n = 3. Statistical significance was determined by two-tailed Student's t-test. Source data are provided as a Source Data file.

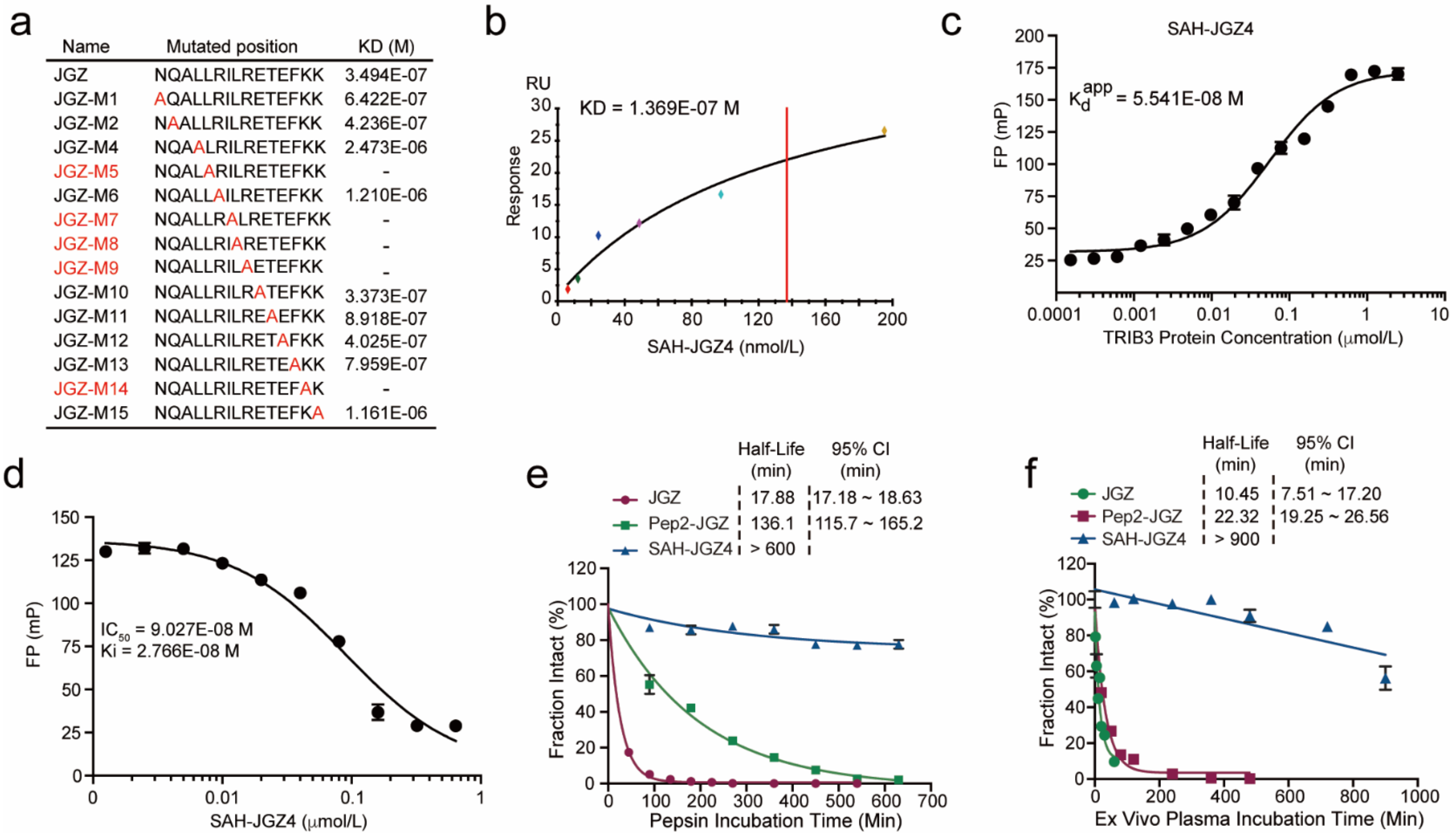
a
Name Mutated position KD (M)
JGZ NQALLRILRETEFKK 3.494E-07
JGZ-M1 AQALLRILRETEFKK 6.422E-07
JGZ-M2 NAALLRILRETEFKK 4.236E-07
JGZ-M4 NQAALRILRETEFKK 2.473E-06
JGZ-M5 NQALARILRETEFKK -
JGZ-M6 NQALLAILRETEFKK 1.210E-06
JGZ-M7 NQALLRALRETEFKK -
JGZ-M8 NQALLRIARETEFKK -
JGZ-M9 NQALLRILAETEFKK -
JGZ-M10 NQALLRILRATEFKK 3.373E-07
JGZ-M11 NQALLRILREAEFKK 8.918E-07
JGZ-M12 NQALLRILRETAFKK 4.025E-07
JGZ-M13 NQALLRILRETEAKK 7.959E-07
JGZ-M14 NQALLRILRETEFAK -
JGZ-M15 NQALLRILRETEFKA 1.161E-06
b
RU
KD = 1.369E-07 M
Response
SAH-JGZ4 (nmol/L)
c
SAH-JGZ4
K$_d^{app}$ = 5.541E-08 M
FP (mP)
TRIB3 Protein Concentration (μmol/L)
d
IC$_{50}$ = 9.027E-08 M
Ki = 2.766E-08 M
FP (mP)
SAH-JGZ4 (μmol/L)
e
Half-Life (min) 95% CI (min)
JGZ 17.88 17.18 ~ 18.63
Pep2-JGZ 136.1 115.7 ~ 165.2
SAH-JGZ4 > 600
Fraction Intact (%)
Pepsin Incubation Time (Min)
f
Half-Life (min) 95% CI (min)
JGZ 10.45 7.51 ~ 17.20
Pep2-JGZ 22.32 19.25 ~ 26.56
SAH-JGZ4 > 900
Fraction Intact (%)
Ex Vivo Plasma Incubation Time (Min)

Supplementary Figure 5 Alanine screening of JGZ peptide and the analysis of SAH-JGZ4 physiochemical properties.

a, Each amino acid of JGZ was substituted with alanine. Kinetic interactions of mutated peptides with TRB3 were determined by SPR analyses. **b,** Steady-State-based affinity determination of SAH-JGZ4. **c,** FP saturation binding curves of FAM-SAH-JGZ4 (10 nM) with increasing concentrations of TRIB3. **d,** FP competitive binding curve. Increasing concentrations of unlabeled peptide SAH-JGZ4 were added to the mixtures of FAM-SAH-JGZ4 (10 nM) and the GST-TRIB3 recombinant protein (220 nM). The FP values were detected 2 hr later. **e,** Pepsin resistance profiles of JGZ, Pep2-JGZ and SAH-JGZ4. The reaction productions were evaluated by LC/MS analysis. **f,** The stability of JGZ, Pep2-JGZ and SAH-JGZ4 in rat plasma was determined by RP-HPLC analyses. Data in c-f are means ± SEM of 3 independent assays. Source data are provided as a Source Data file.

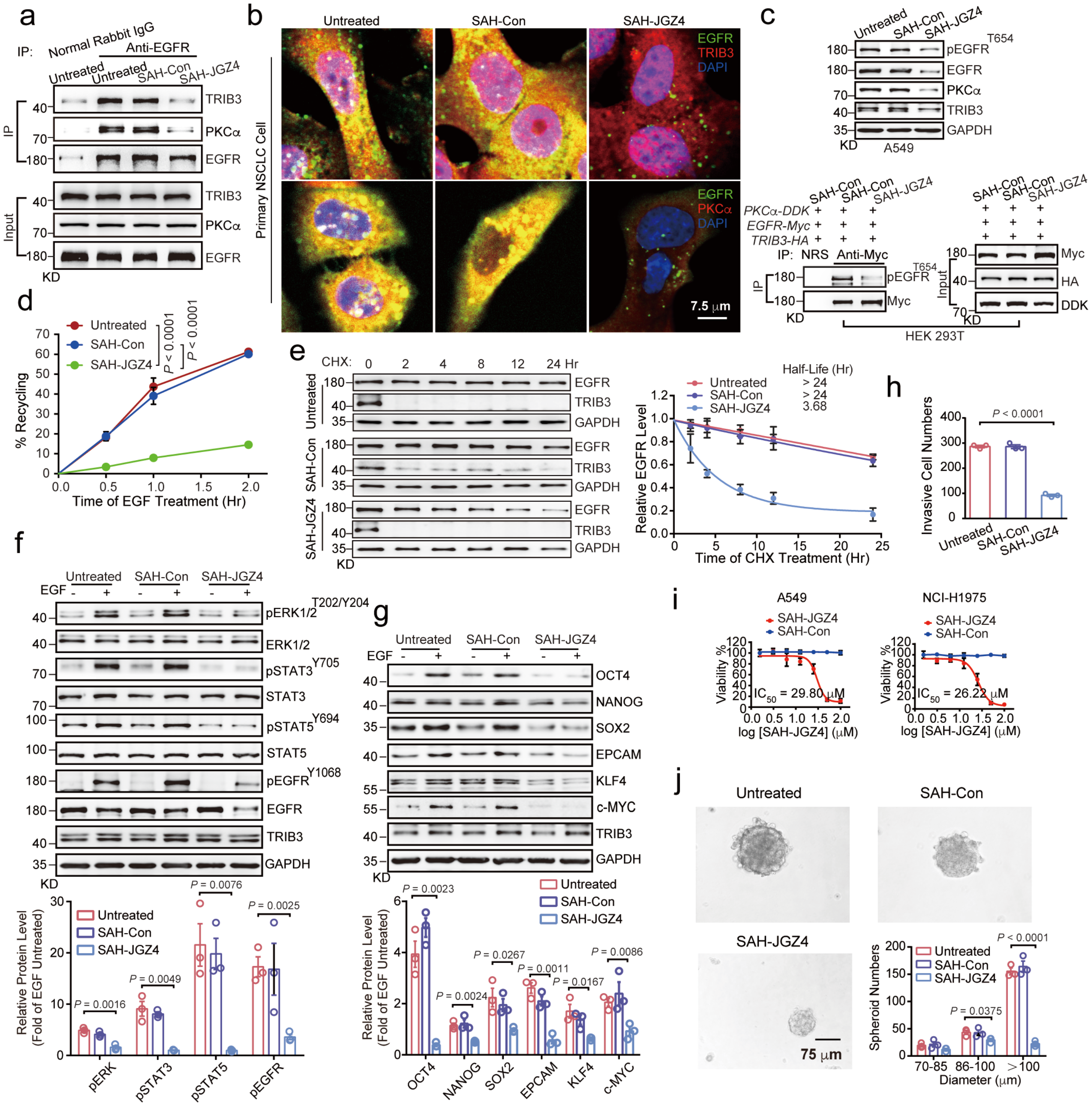

a
IP: Normal Rabbit IgG Anti-EGFR
Untreated Untreated SAH-Con SAH-JGZ4
TRIB3
PKCα
EGFR
IP
Input
KD
b
Untreated SAH-Con SAH-JGZ4
Primary NSCLC Cell
EGFR TRIB3 DAPI
EGFR PKCα DAPI
7.5 μm
c
Untreated SAH-Con SAH-JGZ4
pEGFR T654
EGFR
PKCα
TRIB3
GAPDH
KD A549
PKCα-DDK
EGFR-Myc
TRIB3-HA
IP: NRS Anti-Myc
Input
Myc
HA
DDK
HEK 293T
d
Untreated
SAH-Con
SAH-JGZ4
P < 0.0001
% Recycling
Time of EGF Treatment (Hr)
e
CHX: 0 2 4 8 12 24 Hr
Half-Life (Hr)
Untreated > 24
SAH-Con > 24
SAH-JGZ4 3.68
Relative EGFR Level
Time of CHX Treatment (Hr)
h
Invasive Cell Numbers
P < 0.0001
f
EGF
pERK1/2 T202/Y204
ERK1/2
pSTAT3 Y705
STAT3
pSTAT5 Y694
STAT5
pEGFR Y1068
Relative Protein Level (Fold of EGF Untreated)
P = 0.0016
P = 0.0049
P = 0.0076
P = 0.0025
pERK pSTAT3 pSTAT5 pEGFR
g
OCT4
NANOG
SOX2
EPCAM
KLF4
c-MYC
P = 0.0023
P = 0.0024
P = 0.0267
P = 0.0011
P = 0.0167
P = 0.0086
i
A549
NCI-H1975
Viability %
IC50 = 29.80 μM
IC50 = 26.22 μM
log [SAH-JGZ4] (μM)
j
Untreated SAH-Con SAH-JGZ4
75 μm
Spheroid Numbers
P = 0.0375
P < 0.0001
70-85 86-100 >100
Diameter (μm)

Supplementary Figure 6 Disturbing the TRIB3-EGFR interaction induces tumor suppression in vitro.

a, A549 cells were treated with 5 μM of the indicated peptides for 12 hr. Cell extracts were IP with anti-EGFR Ab and blotted with anti-TRIB3 and anti-PKCα Abs. Data are representatives of 3 independent assays. **b,** Primary NSCLC cells were treated with 5 μM of the indicated peptides for 24 hr. Immunostaining images show the co-localization of indicated proteins. Data are representatives of 3 independent assays. **c,** Top: A549 cells were treated with 5 μM of the indicated peptides for 24 hr. Immunoblots show the expression of the indicated proteins. Bottom: HEK 293T cells were transfected with indicated plasmids and treated with 5 μM of the indicated peptides for 24 hr. Cell extracts were IP with anti-Myc Ab and blotted with indicated Abs. Data are representatives of 3 independent assays. **d,** A549 cells were pretreated with 5 μM of the indicated peptides for 24 hr, then the recycling of EGFR was determined by ELISA analysis. **e,** A549 cells were pretreated with 5 μM of the indicated peptides for 12 hr. The cells were then incubated with CHX (10 μg ml^{-1}) for indicated times. The indicated proteins were detected by IB. **f,** A549 cells were treated with 5 μM of the indicated peptides for 24 hr and stimulated with EGF (100 ng ml^{-1}) for 1 hr. The indicated proteins were detected by IB (n =3). **g,** A549 cells were treated with 5 μM of the indicated peptides for 24 hr and stimulated with EGF (100 ng ml^{-1}) for 1 hr. The stem cell markers were detected by IB (n =3). **h,** The invasive capacities of A549 cells treated with indicated peptides were evaluated with transwell assays. **i,** NSCLC cell lines were treated with SAH-JGZ4 or SAH-Con at indicated concentrations for 72 hr. Cell viability was assessed by CCK-8 assay. **j,** Representative

micrographs and statistical data of oncosphere growth of A549 cells treated with indicated peptides. Data represent means ± SEM of 3 assays. Statistical significance among groups was determined by one-way ANOVA test. Source data are provided as a Source Data file.

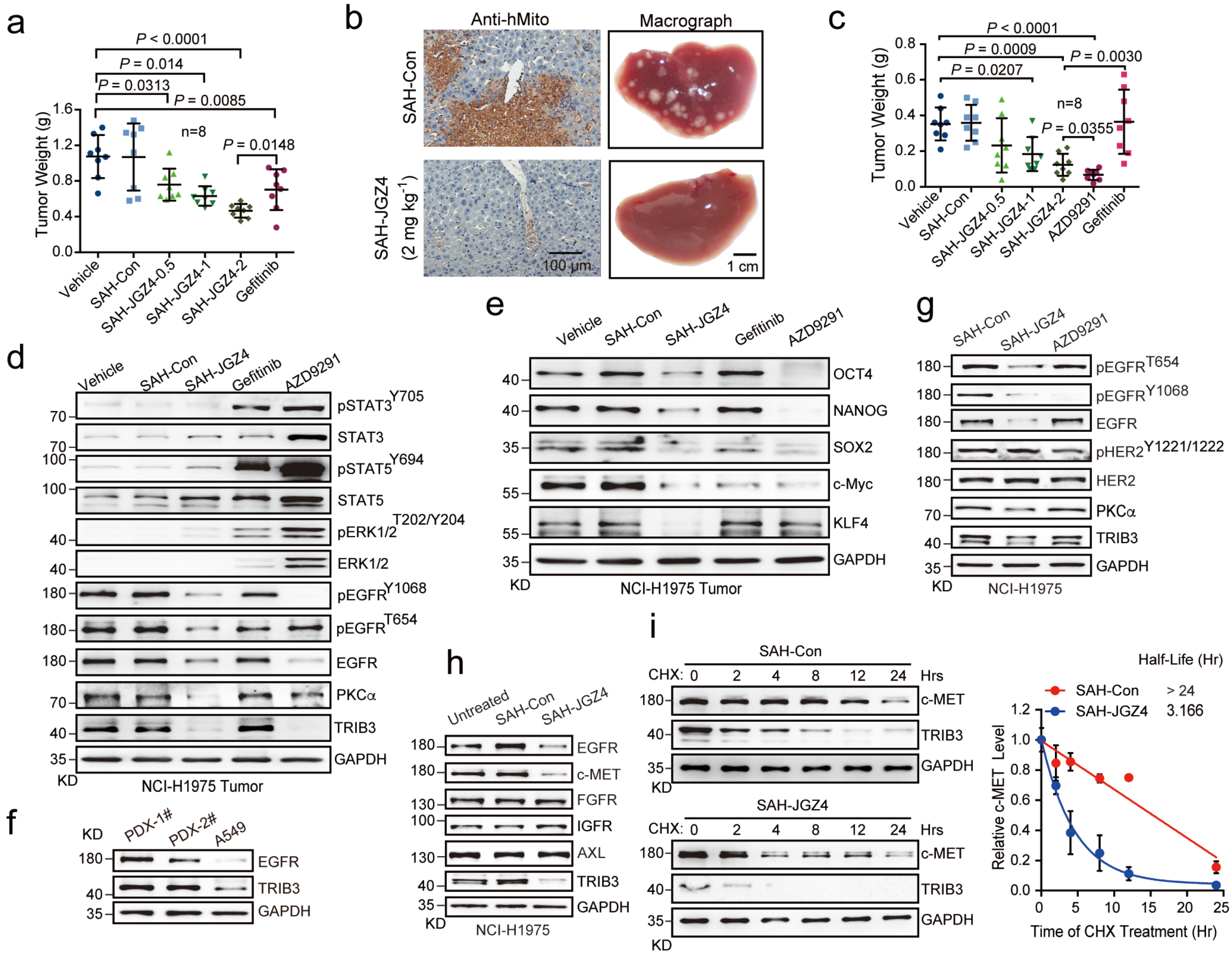
a
P < 0.0001
P = 0.014
P = 0.0313
P = 0.0085
P = 0.0148
n=8
Tumor Weight (g)
Vehicle
SAH-Con
SAH-JGZ4-0.5
SAH-JGZ4-1
SAH-JGZ4-2
Gefitinib
b
Anti-hMito
Macrograph
SAH-Con
SAH-JGZ4 (2 mg kg-1)
100 μm
1 cm
c
P < 0.0001
P = 0.0009
P = 0.0207
P = 0.0030
P = 0.0355
n=8
Tumor Weight (g)
AZD9291
d
pSTAT3 Y705
STAT3
pSTAT5 Y694
STAT5
pERK1/2 T202/Y204
ERK1/2
pEGFR Y1068
pEGFR T654
EGFR
PKCα
TRIB3
GAPDH
KD
NCI-H1975 Tumor
e
OCT4
NANOG
SOX2
c-Myc
KLF4
GAPDH
NCI-H1975 Tumor
g
pHER2 Y1221/1222
HER2
NCI-H1975
f
PDX-1#
PDX-2#
A549
h
Untreated
c-MET
FGFR
IGFR
AXL
NCI-H1975
i
CHX:
Hrs
Half-Life (Hr)
SAH-Con > 24
SAH-JGZ4 3.166
Relative c-MET Level
Time of CHX Treatment (Hr)

Supplementary Figure 7 SAH-JGZ4 treatment inhibits tumor progression of NSCLC.

a, Quantified tumor weight from mice subcutaneously inoculated with A549 cells under indicated treatment after tumor inoculation. Data are means ± SEM, n = 8. **b,** Representative graphs of liver and immunohistochemical staining of the liver sections with anti-hMitochondria Ab from mice as described in (a). **c,** Quantified tumor weight from mice subcutaneously inoculated with NCI-H1975 cells under indicated treatment after tumor inoculation. Data are means ± SEM, n = 8. **d,** EGFR and its signaling proteins in xenograft tumors from NCI-H1975 inoculated mice were detected by IB. **e,** Stem cell markers in xenograft tumors from NCI-H1975 inoculated mice mice were detected by IB. **f,** Expressions of TRIB3 and EGFR were detected by IB in tumor samples from PDX mice. **g,** NCI-H1975 cells were treated with 5 μM of the indicated peptides or 0.5 μM AZD9291for 24 hr. The expression of indicated proteins was detected with IB. **h,** NCI-H1975 cells were treated with 5 μM of the indicated peptides for 24 hr. The expression of several receptor tyrosine kinases was detected with IB. **i,** NCI-H1975 cells were pretreated with 5 μM of the indicated peptides for 12 hr. The cells were then incubated with CHX (10 μg ml^{-1}) for indicated times. The indicated proteins were detected by IB. Data are means ± SEM of 3 independent assays. Data in d-h are representatives of 3 independent assays. Statistical significance between two groups was determined by two-tailed Student's t-test; Statistical significance among groups was determined by one-way ANOVA test. Source data are provided as a Source Data file.

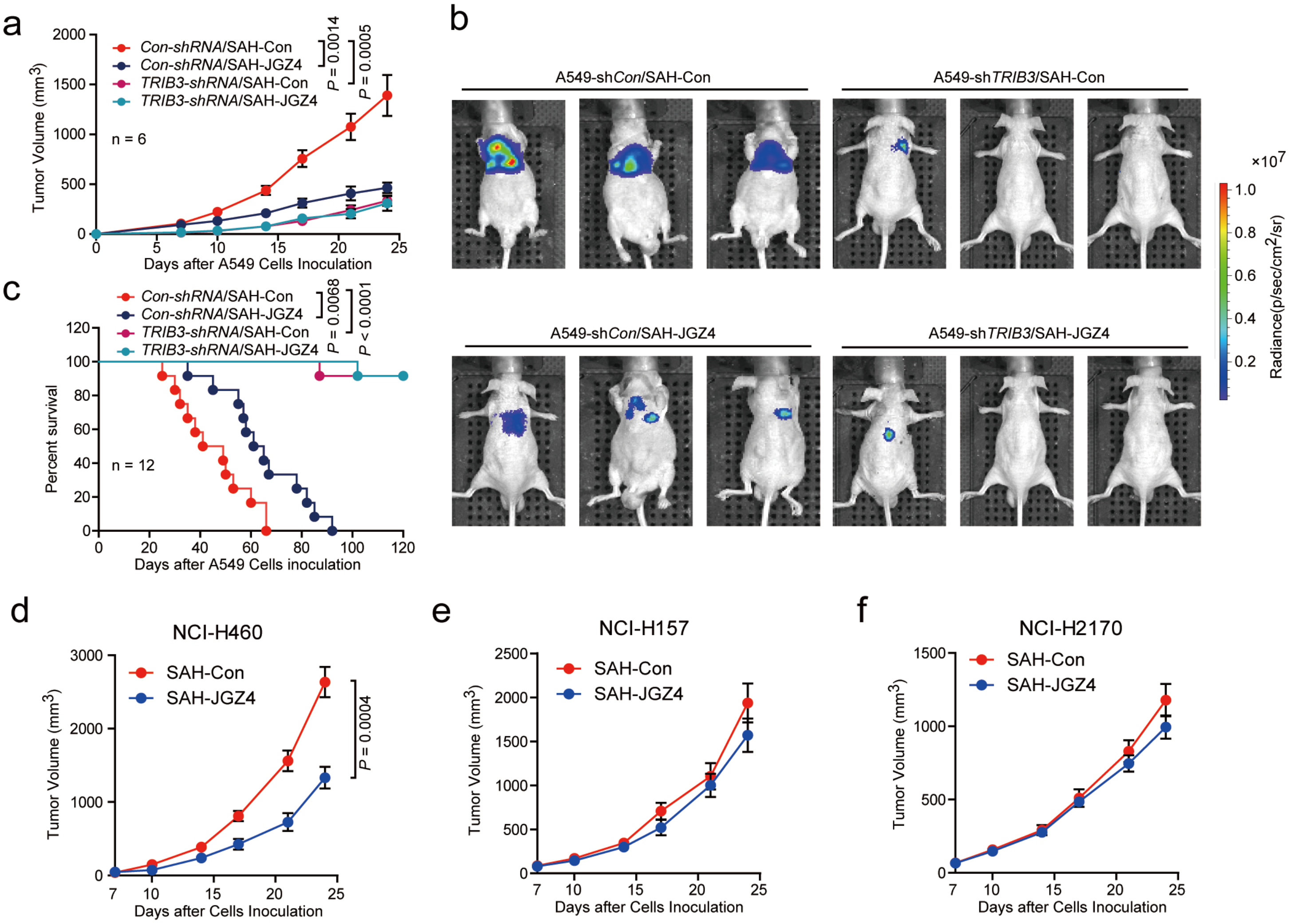
a
Con-shRNA/SAH-Con
Con-shRNA/SAH-JGZ4
TRIB3-shRNA/SAH-Con
TRIB3-shRNA/SAH-JGZ4
P = 0.0014
P = 0.0005
n = 6
Tumor Volume (mm3)
Days after A549 Cells Inoculation
b
A549-shCon/SAH-Con
A549-shTRIB3/SAH-Con
A549-shCon/SAH-JGZ4
A549-shTRIB3/SAH-JGZ4
×10^7
Radiance(p/sec/cm2/sr)
c
Con-shRNA/SAH-Con
Con-shRNA/SAH-JGZ4
TRIB3-shRNA/SAH-Con
TRIB3-shRNA/SAH-JGZ4
P = 0.0068
P < 0.0001
n = 12
Percent survival
Days after A549 Cells inoculation
d
NCI-H460
SAH-Con
SAH-JGZ4
P = 0.0004
Tumor Volume (mm3)
Days after Cells Inoculation
e
NCI-H157
SAH-Con
SAH-JGZ4
Tumor Volume (mm3)
Days after Cells Inoculation
f
NCI-H2170
SAH-Con
SAH-JGZ4
Tumor Volume (mm3)
Days after Cells Inoculation

Supplementary Figure 8 High expression of both TRIB3 and EGFR determines the sensitivity of cancer cells to SAH-JGZ4 treatment.

a, BALB/c nude mice subcutaneously inoculated with A549 cells with or without TRIB3 knocking down (1.0×10^5/mouse) were treated with SAH-Con or SAH-JGZ4 (2 mg kg^{-1}) twice a week. Data are tumor growth curves with means ± SEM at indicated times, n = 6. **b,c,** BALB/c nude mice intravenously inoculated with luciferase-labeled A549 cells with or without TRIB3 knocking down (2.0×10^6/mouse) were treated with SAH-Con or SAH-JGZ4 (2 mg kg^{-1}) twice a week. Data shown are representative bioluminescence imaging of mice under different treatment (b) and Kaplan-Meier survival curves for indicated groups of mice (c), n =12. **d-f,** Tumor growth curves of NCI-H460 (d), NCI-H157 (e) and NCI-H2170 (f) cells under SAH-Con or SAH-JGZ4 treatment (2 mg kg^{-1}, twice a week). Data are means ± SEM, n = 6. Statistical significance between two groups was determined with two-tailed Student's t-test. Statistical significance among groups was determined by one-way ANOVA test. Source data are provided as a Source Data file.

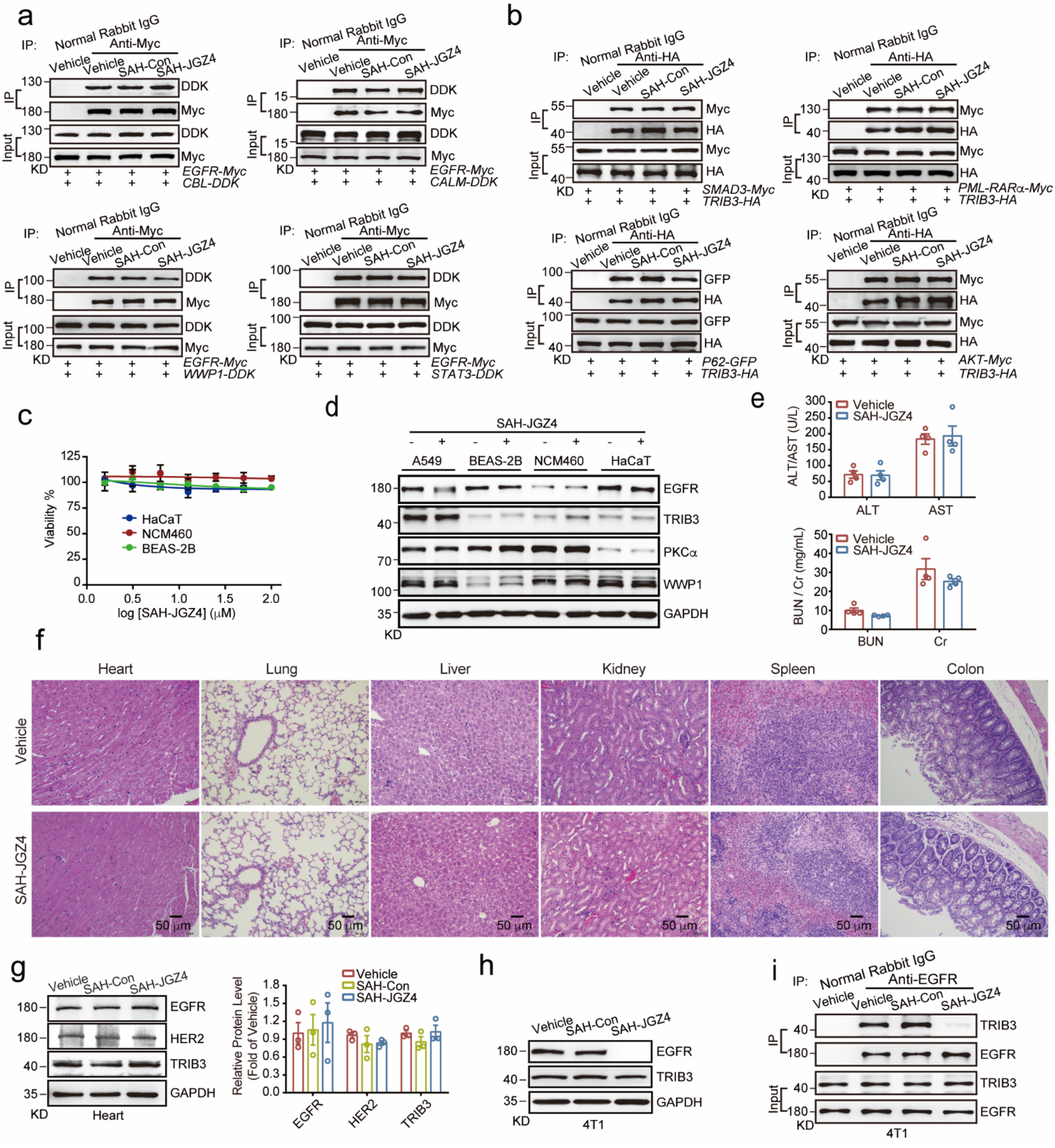

a
IP: Normal Rabbit IgG
Anti-Myc
Vehicle
Vehicle
SAH-Con
SAH-JGZ4
IP
Input
KD
DDK
Myc
130
180
15
100
EGFR-Myc
CBL-DDK
CALM-DDK
WWP1-DDK
STAT3-DDK
b
IP: Normal Rabbit IgG
Anti-HA
Myc
HA
GFP
55
40
SMAD3-Myc
TRIB3-HA
PML-RARα-Myc
P62-GFP
AKT-Myc
c
Viability %
log [SAH-JGZ4] (μM)
HaCaT
NCM460
BEAS-2B
125
100
75
50
25
0
0.0
0.5
1.0
1.5
2.0
d
SAH-JGZ4
A549
BEAS-2B
NCM460
HaCaT
EGFR
TRIB3
PKCα
WWP1
GAPDH
e
ALT/AST (U/L)
Vehicle
SAH-JGZ4
ALT
AST
BUN / Cr (mg/mL)
BUN
Cr
f
Heart
Lung
Liver
Kidney
Spleen
Colon
Vehicle
SAH-JGZ4
50 μm
g
Vehicle
SAH-Con
SAH-JGZ4
EGFR
HER2
TRIB3
GAPDH
Heart
Relative Protein Level (Fold of Vehicle)
h
4T1
i
IP: Normal Rabbit IgG
Anti-EGFR

Supplementary Figure 9 SAH-JGZ4 demonstrates an ideal target specificity and safety.

a, CO-IP assay evaluated the effect of SAH-JGZ4 on the interactions of EGFR/CBL, EGFR/CALM, EGFR/WWP1 and EGFR/STAT3. HEK 293T cells were transfected with indicated plasmids and treated with 5 μM of SAH-Con or SAH-JGZ4. Cell extracts were IP with normal rabbit IgG or anti-Myc Ab and blotted with anti-DDK antibody. **b,** CO-IP assay evaluated the effect of SAH-JGZ4 on the interactions of SMAD3/TRIB3, PML-RARα/TRIB3, P62/TRIB3 and AKT/TRIB3. HEK 293T cells were transfected with indicated plasmids and treated with 5 μM of SAH-Con or SAH-JGZ4. Cell extracts were IP with normal rabbit IgG or anti-HA Ab and blotted with indicated Abs. **c,** Cell viability of indicated cells treated with increasing concentrations of SAH-JGZ4. Data are means ± SEM of 3 independent assays. **d,** The effect of SAH-JGZ4 on the expression of indicated proteins in different cell lines. **e,** Serum alanine aminotransferase (ALT), aspartate aminotransferase (AST), Blood urea nitrogen (BUN) and Creatinine (Cr) were measured in the serum from healthy mice treated with SAH-JGZ4 (2 mg kg^{-1}) twice a week for 4 weeks. Data are presented as mean ± SEM (n=4 mice per group). **f,** Healthy BALB/c nude mice (6 weeks) were treated with or without SAH-JGZ4 (2 mg kg^{-1}) twice a week for 4 weeks. Data shown are representative H&E staining images of heart, lung, liver, kidney, spleen and colon (n = 6). **g,** The expressions of EGFR, HER2 and TRIB3 in the heart from mice treated with different peptides. Data are means ± SEM of 3 independent assays. **h,** The effect of SAH-JGZ4 on the expression of EGFR in 4T1 cells. **i,** The effect of SAH-JGZ4 on the interaction of EGFR/TRIB3 in 4T1 cells. CALM: Calmodulin. Data in a, b, d, h and i are representatives of 3 independent assays. Source data are provided as a Source Data file.

Supplementary Tables

Supplementary Table 1. Summary of mass spectrometry (MS) analysis of E3 ubiquitin ligases related to EGFR.

Band (KD)	Identified Proteins	Peptides	Description	Gene ID
105	WWP1	NQLQGAMQQFNQR<br>VYYVDHNTR<br>TQGLQNEEPLPEGWEIR<br>TYYVDHNTR<br>GGPQIAYER<br>SILEPDTSNSR<br>NFEQWQSQR<br>VYFVNHNTK<br>TTTWQRPTMESVR<br>TTTWERPQPLPPGWER<br>YFVDHNTR	WW Domain Containing E3 Ubiquitin Protein Ligase 1	11059
34	RNF126	DDYALGER	Ring Finger Protein 126	55658
118	RNF213	TQTGHVLGNPQR<br>LNDTVTVR	Ring Finger Protein 213	57674
72	TRIM32	ITSLTQLTDNLTVLK<br>AAAASHLNLDALR	RING-Type E3 Ubiquitin Transferase TRIM32	22954
32	CHIP	YMADMDELFSQVDEK<br>LNFGDDIPSALR<br>WNSIEER<br>ALELDGQSVK<br>SPLTQEQLIPNLAMK<br>DIEEHLQR	RING-Type E3 Ubiquitin Transferase CHIP	10273

Supplementary Table 2. Clinical information of lung cancer samples used for Figure 7l.

Sample NO.	Age	Gender	Pathologic Type	EGFR alteration	KRAS alteration
1	33	M	Adenocarcinoma	Wild type	G12D mutation
2	45	M	Adenocarcinoma	19DEL	Wild type
3	49	F	Adenocarcinoma	Wild type	Wild type

Supplementary Table 3. Clinical information of lung cancer samples used for Figure 8d-m.

Sample NO.	Age	Gender	Pathologic Type	EGFR alteration	KRAS alteration
PDX-1# ("Sample 1" in Supplementary Table 2)	33	M	Adenocarcinoma	Wild type	G12D mutation
PDX-2#	62	M	Adenocarcinoma	Wild type	Wild type

Supplementary Methods

Plasmid Construction

pcDNA6A-EGFR WT, pcDNA6A-EGFR ECD (1-644), pcDNA6A-EGFR ICD (645-1186) plasmids are kind gifts from Prof. Mien-Chie Hung (University of Texas MD Anderson Cancer Center, Houston, TX, United States). The truncations of EGFR, JM (amino acids 645-695), TK (amino acids 689-955), CT (amino acids 956-1186) were constructed into pEGFP-C1 vector by standard subcloning. HA-tagged or GFP-tagged TRIB3 (amino acids 1-359) and its truncations, M1 (amino acids 1-179), M2 (amino acids 180-359), M3 (amino acids 72-315), M4 (amino acids 180-315) and M5 (amino acids 72-359, Δ180-315) plasmids were constructed into pcDNA 3.1-HA or pEGFP-C1 vector by standard subcloning. To establish TRIB3-shRNA1 resistant expression construct (TRIB3$^{si\text{-}Ris}$), R34 codon was mutated from CGA to AGG. EGFR mutants (T654A, T654D, K652R, K684R, K689R, K690R, K692R and T654D/K689R) and JM-GFP mutants (K689, the four Lysine sites except for Lysine 689 in JM-GFP were mutated to arginine; K689/T654A) were established using Fast Mutagenesis System (TransGen Biotech, Beijing, China). EGFRvIII plasmid was constructed into pCMV6-Entry vector by standard subcloning. Ubiquitin (Ub) and Ub^{K63R} sequences were synthesized commercially and constructed into pFLAG-CMV2 vector to establish the Ub-DDK and Ub^{K63R}-DDK expressing plasmids. PKCα-DDK and WWP1-HA plasmids were purchased from Sino Biological Inc. (Beijing, China). The Halo-GFP-Mito plasmid was designed and constructed as previously reported[32]. Briefly, the C terminus of Haloenzyme–GFP was fused with the mitochondrial outer membrane targeting domain comprising the C-terminal 47 amino acids of

the Listeria monocytogenes ActA gene, which anchors Halo-GFP to the outer mitochondrial membrane, oriented in the cytosol.

Human Tissue Microarray

Paired cancer and adjacent non-cancer paraffin tissue sections (HLug-Squ150Sur-01 for Lung Squamous Carcinoma and HLug-Ade180Sur-02 for lung adenocarcinoma) were purchased from Shanghai Outdo Biotech (Shanghai, China).

Quantitative Real-time PCR and RNA Interference

Total RNA was extracted using TransZol UP (TransGen Biotech, Beijing, China) following the manufacturer's instructions. Reverse transcription of the total cellular RNA was carried out using oligo (dT) primers and M-MLV reverse transcriptase (Promega, Madison, USA). qPCR was performed using the KAPA SYBR FAST qPCR Master Mix (2×) Kit (Kappa Biosystem, USA) according to the manufacturer's instructions. qPCR primers were as follows: TRIB3 forward, 5'-TCAAGCTGTGTCGCTTTGTC-3'; TRIB3 reverse, 5'-TGTCCCACAGGGAAT CATCT-3'; EGFR forward, 5'-AACTGTGAGGTGGTCCTTG G-3'; EGFR reverse, 5'-GTT GAGGGCAATGAGGACAT-3'; Actin forward, 5'-AGAGCTACGAGCTGCCTGAC-3'; Actin reverse, 5'-AGCACTGTGTTGGCGTACAG-3'. PKCα forward, 5'-ACCATGGCTGA CGTTTTCCC-3'; PKCα reverse, 5'-TTGTGGTCCTTCACCTCGTG-3'; CBL forward, 5'-TGCTTCTCTCCCTCG CTCG-3'; CBL reverse, 5'-CCCCAGAGCTCTTCTTCACG-3'; CIN85 forward, 5'-CTCCCGGGGAAGTCATTCATC-3'; CIN85 reverse, 5'-GTCCGCTCAG AAGACTGGAG-3'. *WWP1* siRNAs (GTGCCAGTCTAATGCACTA) was produced by

RIBOBIO (Guangzhou, China) and transfected using Lipofectamine RNA interference MAX Transfection Reagent (Life Technologies, CA, USA) according to the manufacturer's instructions.

RNA Microarray Assay

Total RNA of A549 cells stably expressed with Control-shRNA, TRIB3- shRNA1, and TRIB3-shRNA2 were obtained. The RNA quantity and quality were measured by NanoDrop ND-1000. RNA microarrays were performed by Compass Biotechnology Co., Ltd (Beijing, China) using Affymetrix Human 2.0 ST Gene Expression Array (Thermo Fisher Scientific, USA) in compliance to MIAME guidelines. The GEO accession number is GSE103891.

Human Proteome Microarray Assay

The HuProt microarray (CDI Laboratories, Inc.) composed of ~20000 human full-length proteins with N-terminal glutathione S-transferase (GST) tags was used to identify TRIB3 interactors. The HuProt microarray assay was performed by Wayen Biotechnologies Inc. (Shanghai, China) according to the following procedure. Human Proteome microarrays (HuProt^{TM} 20K) were blocked with blocking buffer (1% BSA in 0.1% Tween 20, TBST) for 1 hr at room temperature with gentle agitation. TRIB3 protein (Sino Biological Inc., Beijing, China) was labeled with biotin by the Antibody Array Assay Kit (Full moon Biosystems, Sunnyvale, CA), then diluted to 0.01 mg ml^{-1} in blocking buffer and incubated on the blocked proteome microarray at room temperature for 1 hr. The microarrays were washed for 5 min 3 times with TBST, incubated with streptavidin-Cy5 at 1:1,000 dilution (Thermo Fisher

Scientific, USA) for 1 hr at room temperature and underwent three more 5-min washes. The microarrays were spun dry at 1,500 rpm for 3 min and subjected to scanning with a Genepix 4000B (Axon Instruments, Sunnyvale, CA) in order for results to be visualized and recorded. A GenePix Pro 6.0 was used for data analysis.

***In vitro* generation of patient-derived lung cancer organoids**

Primary lung cancer fragments were minced and dissociated in digestion buffer containing collagenase (Sigma, C5138) and hyaluronidase Type IV (Sigma, H3884) for 3 hr at 37℃. The cell pellet was obtained after centrifuge at 530 g for 5 min and suspended again in RMPI-1640 medium. Organoid fraction was collected by centrifuging at 530 g for 10 sec for 3-5 times. The collected pellet was carefully resuspended in growth factor reduced matrigel (BD Biosciences, 354230). About 80 µl matrigel/organoid suspension containing 150 organoids were allowed to solidify on pre-warmed 12-well plate at 37℃ for 30 min. Upon completed gelation, 1 ml of lung cancer organoid medium was added to each well and cultured in the cell incubator. The medium was changed every 3 days and images were captured using an Olympus CKX41 microscope at day 7.

Generation of PDX animal models

Fresh lung cancer tissues were spliced into small fragments (1-3 mm^3) in the medium. The tissue fragments were suspended in diluted Matrigel (Corning, 354248) 1:1 with PBS, and subcutaneously implanted into NCG/NSG (NOD-$Prkdc^{scid}Il2rg^{null}$) mice. The tumors were passaged to a next generation of mice when their volumes were growing to a certain size.

Murine Xenograft Model

For the subcutaneous tumor model, A549 (1.0×10^5), NCI-H1975 (3.0×10^5) or patient-derived NSCLC cancer cells (4.0×10^5) were injected subcutaneously into the right flank of each mouse to establish the NSCLC xenograft model. When average tumor volume reached about 100 mm^3, mice were randomized into various groups for different sets of experiments. Tumor growth was monitored twice weekly with a caliper. Tumor volume (T_V) was calculated using the formula: $T_V=0.5\times L\times W^2$.

To explore the role of SAH-JGZ4 in the treatment of tumor *in vivo*, we used A549 cells (1.0×10^5) in the xenograft experiments. There were six groups: i) Saline (Vehicle); ii) SAH-Control peptide (SAH-JGZ1: $NQ_{S5}LLR_{S5}LRETEFKK$, without binding ability with TRIB3); iii) low-dose SAH-JGZ4 (0.5 mg kg^{-1}); iv) medium-dose SAH-JGZ4 (1 mg kg^{-1}); v) high-dose SAH-JGZ4 (2 mg kg^{-1}); vi) Gefitinib (6.25 mg kg^{-1}). Mice received intraperitoneal injection with the above mentioned peptides twice a week or were given Gefitinib by gavage once per day for three weeks.

In the following experiments, we used NCI-H1975 (3.0×10^5) to establish the xenograft models. There were seven groups: i) Saline (Vehicle); ii) SAH-Control peptide (2 mg kg^{-1}); iii) low-dose SAH-JGZ4 (0.5 mg kg^{-1}); iv) medium-dose SAH-JGZ4 (1 mg kg^{-1}); v) high-dose SAH-JGZ4 (2 mg kg^{-1}); vi) Gefitinib (6.25 mg kg^{-1}); vii) AZD9291 (5 mg kg^{-1}). Mice received intraperitoneal injection with above mentioned peptides twice a week; mice received Gefitinib or AZD9291 by gavage once per day for four weeks.

To evaluated the role of SAH-JGZ4 in the improvement of survival rate of tumor-bearing mice, we established the lung orthotopic implantation model by inoculating luciferase-labeld A549 cells (3.0×10^4) into the lung parenchyma of each mouse. There were two groups: i) SAH-Control peptide (2 mg kg^{-1}) and ii) SAH-JGZ4 (2 mg kg^{-1}). Mice received intraperitoneal injection with the peptides twice a week. *In vivo* animal imaging was also performed to monitor the development of A549 cells in BALB/c nude mice treated with peptides through the IVIS Spectrum optical imaging system 3 days and one month after inoculation.

To evaluate the tumor-initiating-inhibiting effect of SAH-JGZ4, A549 cells (1.0×10^5) were subcutaneously injected into the right flank of of each mouse. One week after tumor inoculation, mice were peritoneally treated with the SAH-JGZ4 (2 mg kg^{-1}) or SAH-Control peptide (2 mg kg^{-1}) twice a week for 4 weeks. Mice were then sacrificed and tumors were dissected. Tumor tissues were dissociated into single-cell suspension with the Tumor Dissociation Kit (Miltenyi Biotec Inc, Bergisch Gladbach, Germany) according to the manufacturer's instructions. The Mouse Cell Depletion Kit (Miltenyi Biotec Inc, Bergisch Gladbach, Germany) was used for the enrichment of untouched human cells. After cell counting, different number of cells (30, 100, 300, 1,000, 3,000) were injected respectively into the right flank of each mouse. Tumor growth was monitored for 8 weeks. Frequency of tumorigenic cell and probability estimates were computed using Extreme Limiting Dilution Analysis (ELDA) software (http://bioinf.wehi.edu.au/software/elda/)[1].

To evaluate the combinational therapeutic effect of SAH-JGZ4 with chemotherapies in NSCLC PDX models, we followed the standard protocol for PDX transplantation, maintenance and digestion of the tumors. Lung cancer cells were isolated from PDX mice by using the Tumor Dissociation Kit human (Miltenyi Biotec, 130-095-929) and subcutaneously injected into the right flank of of NCG mouse (4.0×10^5). One week after tumor inoculation, mice were treated with SAH-JGZ4 (2 mg kg^{-1}, twice a week), with carboplatin (30 mg kg^{-1}, once a week) or pemetrexed (100 mg kg^{-1}, once a day). Tumor growth was monitored twice weekly with a caliper. Tumor volume (T_V) was calculated using the formula: $T_V=0.5\times L\times W^2$. Mice survival was monitored every day until the total tumor volume reached 2500 mm^3 or when mice showed signs of distress.

To roughly evaluate the SAH-JGZ4 safety, healthy BALB/c nude mice (6 weeks) were i.p. treated with or without SAH-JGZ4 (2 mg kg^{-1}) twice a week for 4 weeks. Mice were then sacrificed and serum were collected for biochemical analysis of alanine aminotransferase (ALT), aspartate aminotransferase (AST), Blood urea nitrogen (BUN) and Creatinine (Cr) by using Chemistry Analyzer TBA-40FR (Toshiba Medical System, Japan).

Generation of Stable Cell Lines

To establish cell populations stably expressing *TRIB3-shRNA1/2*, or the *Control-shRNA*, cells were transfected with *TRIB3-shRNA1/2* or the *Control-shRNA* expressing plasmids. Stable transfectants were selected in medium containing hygromycin. After 2-3 passages in the presence of hygromycin, the cultured cells were used for experiments without cloning.

To establish cells stably expressing TRIB3, TRIB3-HA or empty vector plasmids were transfected respectively into NCI-H157 cells with Lipofectamine® LTX-Plus™ transfection reagent according to the manufacturer's instructions. After 24 hr of transfection, stable transfectants were selected in medium containing G418 (GIBCO, CA, USA) without cloning.

To establish *TRIB3-shRNA* cells stably expressing *TRIB3* or *TRIB3-M5* mutant, *TRIB3-shRNA* cells were transduced with recombinant lentivirus containing siRNA-resistant TRIB3 replacement or *TRIB3-M5* mutant expressing plasmid. To establish *TRIB3-shRNA* cells stably expressing $EGFR^{T654A}$, $EGFR^{T654D}$ or $EGFR^{T654D/689R}$ mutant, *TRIB3-shRNA* cells were transduced with recombinant lentivirus containing $EGFR^{T654A}$, $EGFR^{T654D}$ or $EGFR^{T654D/689R}$ mutant expressing plasmid. Stable transfectants were selected by puromycin (GIBCO, CA, USA). To generate NCI-H157/TRIB3-HA cells with PKCα or WWP1 depletion, H157/TRIB3-HA cells were transduced with recombinant lentivirus containing PKCα-shRNA1 or WWP1-shRNA1 (HanBio CO., Ltd, Shanghai, China). Stable transfectants were selected by puromycin. For bioluminescent tracking, cells were lentivirally infected with a reporter construct encoding green fluorescent protein and firefly luciferase (HanBio CO., Ltd, Shanghai, China). GFP-positive cells were enriched by fluorescence-activated cell sorting. To establish cells stably expressing Halo-GFP-Mito, *Halo-GFP-Mito* plasmid was transfected into A549 cells with Lipofectamine® LTX-Plus™ transfection reagent according to the manufacturer's instructions.

Cell proliferation and Cytotoxicity Assay

CCK-8 was conducted to detect the cell viability in cell proliferation and cytotoxicity assays according to the manufacturer's instructions (Dojindo Inc., Kumamoto, Japan). Cells were plated in 96-well plates, starting at 3,000 cells/well. For cytotoxicity assay, the cells were treated with indicated drugs, then a mixture of 10 µl CCK-8 solution and 90 µl complete culture medium was added into each well. After 1 hr of incubation, the absorbance was measured at 450 nm using a microplate reader.

Analysis of cell invasion

For transwell invasion assays, the transwell chambers (Merck Millipore, Darmstadt, Germany) were precoated with 10 µg ml^{-1} Fibronectin on the lower surface, and the polycarbonate filter was coated with Matrigel (30 mg per well; BD Matrigel Matrix). The chambers were then inserted in 24-well culture plates. Next, A549 cells were detached and resuspended in serum-free medium before being plated (2×10^5 cells/well) into the upper chamber and allowed to invade for 12 hr. The penetrated cells were fixed with 4% paraformaldehyde, stained with crystal violet staining solution, and counted under an inverted microscope (200× magnification).

Gene Set Enrichment Analysis (GSEA)

Using a gene-set enrichment analysis (GSEA) signal-to-noise ratio ranking metric, we ranked the 24,838 genes by their association with A549 cells (*Control-shRNA vs. TRIB3-shRNA1*). Pre-ranked GSEA was performed using a data set of the EGFR signaling-associated 330 genes (in combination of "REACTOME SIGNALING BY EGFR IN CANCER", "AMIT EGF RESPONSE 480 HELA" and "PID ERBB1 DOWNSTREAM PATHWAY" gene sets;

http://software.broadinstitute.org/gsea/index.jsp) for its association to TRIB3 expression in A549 cell groups mentioned above. GSEA analysis was conducted using MSigDB v6.1. Gene set was considered significant when the false discovery rate (FDR) was less than 0.25.

Immunoblotting, Immunostaining and Immunohistochemistry

Proteins were extracted from cells and tissues using a RIPA lysis buffer (Applygen Technologies Inc. Beijing, China). Equal protein amounts were loaded for SDS-PAGE, followed by transfer to polyvinylidene difluoride membranes, and subjected to immunoblot analysis. Signals were detected by Tanon 5200 chemiluminescent imaging system (Tanon, Shanghai, China). The western blots were scanning densitometry using Gel Pro Analyzer 3.2 and calculated relative to GAPDH. If not specifically indicated, the data was presented as means ± SEMs of 3 independent biological experiments.

For immunofluorescence staining, cells seeded on coverslips were briefly washed with PBS and fixed with 4% buffered paraformaldehyde for 15 min, permeabilized with 0.5% Triton X-100 for 15 min, blocked with 3% BSA for 30 min at 37℃, and stained with specific primary antibodies followed by corresponding secondary antibodies. Nuclei were counterstained with DAPI. Images were captured using a confocal fluorescent microscope (Olympus Microsystems, CA, USA). Quantitative image analysis was performed with the Imaris 9.3.1 software. Pearson's coefficient was use to analyze colocalization between two target proteins.

For immunohistochemistry analysis, the paraffin-embedded tissue sections were de-paraffinized with xylene and hydrated through graded alcohols into water. Antigen retrieval was carried out with a citrate buffer (10 mM sodium citrate buffer, pH 6.0) at sub-boiling temperature for 15 min. The sections were permeabilized with 0.5% Triton-100/PBS for 20 min. Endogenous peroxidase activity was blocked with 3% H_2O_2 solution for 10 min, followed by washing three times with PBS. Blocking buffer (3% BSA/PBS) was added to the sections and incubated for 30 min. Slides were then incubated with indicated primary antibodies at 4℃ overnight. After washing three times, sections were incubated for 30 min with corresponding secondary antibodies at room temperature. Signals were detected with freshly made DAB substrate solution (ZSGB-BIO Company, Beijing, China). Sections were then counterstained with hematoxylin, dehydrated, and mounted with coverslips. Images were captured using Olympus DP72 microscope (Olympus Microsystems, CA, USA) and analyzed by Image-Pro Plus 5.1.

For immunoblotting, the following antibodies were used: anti-TRIB3 (Abcam, ab75846, 1:1000), anti-TRIB3 (ThermoFisher, PA5-15480, 1:1000), anti-EGFR (CST, #4267, 1:1000), anti-GAPDH (ZSGB-BIO, TA-08, 1:2000), anti-Phospho-p44/42 MAPK (Erk1/2) (Thr202/Tyr204) (CST, #4370, 1:1000), anti-p44/42 MAPK (Erk1/2) (CST, #4695, 1:1000), anti-Phospho-Stat3 (Tyr705) (CST, #9145, 1:1000), anti-Stat3 (CST, #9139, 1:1000), anti-Phospho-Stat5 (Tyr694) (CST, #4322, 1:1000), anti-Stat5 (CST, #94205, 1:1000), anti-phospho-EGF Receptor (Tyr1068) (CST, #3777, 1:1000), anti-PKCα (CST, #2056, 1:1000), anti-Anti-phospho-EGFR (Thr654) (Merck, #04-282, 1:500), anti-Myc (MBL, #562, 1:1000),

anti-GFP (MBL, #598, 1:1000), anti-HA (MBL, #561, 1:1000) anti-DDK (MBL, PM020), anti-WWP1 (Abcam, ab43791, 1:1000), anti-K63-linkage Specific Polyubiquitin (CST, #5621, 1:1000), anti-K48-linkage Specific Polyubiquitin (CST, #8081, 1:1000), anti-c-Myc (CST, #18583, 1:1000), anti-Oct4 (CST, #2750, 1:1000), anti-KLF4 (CST, #4038, 1:1000), anti-Nanog (CST, #4903, 1:1000), anti-Sox2 (CST, #14962, 1:1000), anti-Epcam (CST, #14962, 1:1000), anti-c-Met (CST, #8198, 1:1000), anti-AXL (CST, #8661, 1:1000), anti-IGF-I Receptor β (CST, #9750, 1:1000), anti-FGFR1 (CST, #9740, 1:1000), anti-Her2 (CST, #4290, 1:1000); For immunofluorescence and immunohistochemistry, the following antibodies were used: anti-TRIB3 (Abcam, ab137526, 1:100) anti-Rab11 (CST, #5589, 1:100), anti-EGFR (Abcam, ab231, 1:100), anti-EGFR (Santa Cruz, R-1, #sc-101 AF488, 1:100), anti-EGFR (CST, #4267, 1:100), anti-EEA1 (Abcam, #ab70521, 1:100), anti-Lamp1 (Abcam, #ab25630, 1:100), anti-PKCα (CST, #2056, 1:100), anti-PKCα (Abcam, # ab32376, 1:100), anti-PKC (Abcam, #ab31, 1:100), Alexa Fluor™ 555 Phalloidin (Invitrogen, #A34055, 1:200), WWP1 (Abcam, ab227213, 1:100).

Immunoprecipitation and Mass Spectrometry (MS)

Co-IP was performed to verify PPI. In brief, cell lysates were incubated with indicated antibodies and Protein A/G Plus-Agarose (Santa Cruz Biotechnology, TX, USA) at 4℃ overnight. The immunocomplex was washed 4-6 times, and boiled in 2×SDS sample buffer for 5 min. The co-precipitates were resolved using SDS-PAGE and blotted with specific antibodies. The bound proteins were dissolved in SDS sample buffer and analyzed by immunoblotting. Expression of GST fusion proteins was confirmed by SDS PAGE and Coomassie Blue staining.

For protein identification via mass spectrometry, A549 cells were treated with EGF (100 ng ml^{-1}) for 30 min and whole cell lysates were extracted and immunoprecipitated with anti-EGFR antibody using Pierce CO-IP Kit (Thermo Scientific, USA). The eluents were separated on SDS-PAGE gel followed by silver staining; the bands were extracted from the gel and subjected to LC-MS/MS sequencing and data analysis by QLBio Biotechnology Co., Ltd (Beijing, China). In brief, proteins were digested in gel and extracted. The digestion products were separated by a 120/60 min gradient elution at a flow rate 0.600 μL min^{-1} with the EASY-nLC 1000 system which was directly interfaced with the Thermo Orbitrap Fusion mass spectrometer. The mass of peptides was identified by LC-MS/MS Q Exactive^{TM} Hybrid Quadrupole- Orbitrap Mass Spectrometer (Thermo Scientific, USA). Data of MS/MS were searched against the human fasta from UniProt using an in-house Proteome Discoverer (Version PD1.4, Thermo-Fisher Scientific, USA). Peptides only assigned to a given protein group were considered as unique.

Flow cytometry

Live cells were stained with Alexa-Fluro 488 anti-EGFR antibody (Santa Cruz, R-1, #sc-101 AF488, 1:100). Analysis was performed using a PARTEC EPICSXL (Germany) and data was analyzed using FCS Express 6.

Tumor Sphere Forming Assay

Cells were digested and gently suspended into a single cell suspension with the StemXVivo Serum-Free Tumorsphere Media (R&D systems, Minneapolis, USA) containing heparin and Hydrocortisone. 10,000 cells were resuspended in 1 ml completed StemXVivo medium and

transferred to each well of ultralow adhesion 12-well plates. The cell culture plate was then moved into 37℃ cell culture incubator and maintained for 7 days. Cells were fixed with 4% buffered paraformaldehyde for 15 min, permeabilized with 0.5% Triton X-100 for 15 min, and stained with Phalloidin CruzFluor™-555 Conjugate F-Actin for 48 hr. Nuclei were counterstained with DAPI. Images were captured using a confocal fluorescent microscope (Olympus Microsystems, CA, USA).

Surface Plasmon Resonance Analysis

Binding kinetics between TRIB3 and indicated peptides were analyzed by a surface plasmon resonance assay using BIAcore T200 instrument (GE Healthcare, Pittsburgh, USA). The dissociation constant (KD) was calculated according to the BIA-evaluation software.

Fluorescence Polarization Binding Assay and Competition Assay

For the FP saturation binding experiment, 10 nM FAM-labeled SAH-JGZ4 peptide were mixed with increasing concentrations of the TRIB3-GST recombinant protein. After incubation at room temperature for 2 hr, the FP signals were detected by a multifunctional microplate (EnVision, Perkin Elmer) in black 384-well microplates. 480-nm excitation and 535-nm emission filters were used for the measurements. For the competitive binding assay, a mixture containing 10 nM FAM-labeled SAH-JGZ4 peptides and 220 nM TRIB3-GST recombinant protein was incubated with serial dilutions of unlabeled SAH-JGZ4 peptides for 2 hr at room temperature. The IC_{50} values (the concentrations required for 50% displacement of the tracer) and the Ki values of competitive inhibitors were calculated with GraphPad Prism 7.

Circular Dichroism Spectroscopy

Far-UV circular dichroism was performed using a Jasco J-815 spectrometer equipped with a Jasco PTC-423S/15 temperature system (Jasco Corporation, Japan). Buffer sample containing no peptide was subtracted from all spectra to account for any background signal. CD spectra were recorded at 20℃ using a 300-190 nm measurement range, 0.5 nm step resolution, 50 nm min^{-1} scanning speed, 6 accumulations, 1 sec response time, and 1 nm bandwidth. All spectra were the average of 6 accumulations after background subtraction and converted to a uniform scale of molar CD extinction coefficients. The contents of secondary structures were calculated using the built-in algorithms.

Chloroalkane Penetration Assay (CAPA)

A549 cells stably expressing Halo-GFP-Mito were seeded in a 12 well plate. After 24 hr culture, cells were washed with PBS and treated with indicated chloroalkane-tagged peptides in phenol red free Opti-MEM for 4 hr. Then the Medium was aspirated and cells were washed with Opti-MEM for 15 min. Cells were then incubated with 5 μM HaloTag® TAMRA Ligand (Promega) in phenol red free Opti-MEM for 15 min. After washing with PBS, cells were analyzed by fluorescence microplate reader or fluorescence microscopy.

Metabolic stability of peptide

The sample solution consisted of 15 μl peptide solution (1 mM) and 585 μl Tris-HCl (pH = 7.4) buffer. Pepsin solution (4,500 U ml^{-1}, 8 μl) or proteinase K solution (4 ng ml^{-1}, 8 μl) was added to the sample solution and incubated at 25℃ for indicated times. Peptide concentrations were

determined by reversed-phase HPLC (Waters Symmetry 3.5 μm 4.6 × 150 mm C18) and detected by Exactive™ Plus Orbitrap (Thermo Fisher Scientific, USA).

***In vitro* plasma stability assay**

The in vitro stability of the peptide was studied in rat plasma. The rat blood was collected by retroorbital puncture and plasma was harvested after centrifugation (2,500×g, 5 min, 4℃) and stored at -70℃. The reactions were initiated by adding the test peptide to 100 μl of preheated plasma solution to yield a final concentration of 4 mM. The plasma solutions were incubated for indicated times and added 200 μl acetonitrile to deproteinize the plasma. The samples were subjected to vortex mixing for 1 min and centrifugation for 15 min (14,000 rpm, 4℃). Peptide concentration in supernatants was determined by reversed-phase HPLC with electrospray ionization mass spectrometric detection. The values represent the mean of 3 independent assays.

***In vivo* bio-distribution and clearance of peptide**

FAM-SAH-JGZ4 or FAM-Pep2-JGZ peptide were dissolved in PBS and administered to 6 wk old male athymic BALB/c nude mice by bolus tail vein injection (2 mg kg^{-1}). The fluorescence of FAM labeled peptide was monitored by bioluminescence imaging using IVIS (Perkin EImer, OH, USA). Data was analyzed by the built-in software.

Supplementary Notes

Supplementary Note 1. Evaluation of SAH-JGZ4 off-target effects and safety

Off-target effects of SAH-JGZ4 treatment were examined because EGFR was found to interact with several proteins[2, 3, 4], including WWP1, which we identified in this study. The reported binding partners of TRIB3 were also evaluated[5, 6, 7, 8]. SAH-JGZ4 did not affect the interaction of EGFR with Casitas B Lymphoma (CBL), Calmodulin (CALM), STAT3 or WWP1 (Supplementary Fig. 9a). Additionally, SAH-JGZ4 did not disturb the interactions of TRIB3 with SMAD Family Member 3 (SMAD3), Promyelocytic leukemia/Retinoic Acid Receptor alpha (PML-RARα), AKT or p62 (Supplementary Fig. 9b). Side effects or toxicities, such as cutaneous reactions, gastrointestinal toxicity and even heart damage, are unavoidable problems of most EGFR targeted agents[9]. We found that SAH-JGZ4 treatment had no effect on the cell viability of several normal human cell types, such as keratinocytes (HaCaT), colonic mucosal epithelial cells (NCM460), and bronchial epithelial cells (BEAS-2B) (Supplementary Fig. 9c). In fact, these three cell lines expressed much lower levels of TRIB3 than A549 cells (Supplementary Fig. 9d), suggesting that SAH-JGZ4 may have a smaller impact on normal epithelial cells than on cancerous epithelial cells because of the low expression level of TRIB3 in normal cells. *In vivo* studies showed that SAH-JGZ4 treatment did not cause liver or renal function abnormalities in healthy mice (Supplementary Fig. 9e); no obvious histopathological changes in the heart, lungs, liver, kidneys, spleen or colon were found in the SAH-JGZ4-treatment group (Supplementary Fig. 9f). Additionally, SAH-JGZ4 did not cause reductions in EGFR and HER2 expression in the heart (Supplementary Fig. 9g). To exclude the possibility that the lack of toxicity in the mice is due to the inability of this peptide to target mouse EGFR,

4T1 cells (a murine mammary carcinoma cell line with high expression levels of both EGFR and TRIB3) were treated with SAH-JGZ4. It showed that SAH-JGZ4 treatment not only decreased EGFR expression, but also disrupted the interaction between EGFR and TRIB3 in the 4T1 cells (Supplementary Fig. 9h, i), indicating SAH-JGZ4 had a good ability to target mouse EGFR. Together, these data suggest that SAH-JGZ4 is a candidate therapeutic option for EGFR-related NSCLC treatment that functions by promoting EGFR degradation with fewer side effects.

Supplementary Discussion

Here, NCI-H157 and NCI-H1650 cells displayed high baseline levels of EGFR but very low TRIB3 expression, suggesting that high EGFR expression may be regulated through other mechanisms in these two cell lines. ERK activity is known to positively regulate of EGFR transcription via c-jun[10]. NCI-H157 and NCI-H1650 cells may have constitutive activation of ERK activity because they harbor KRAS- or EGFR activating mutations respectively. In our study, high TRIB3 mRNA level did not correlate with the survival of squamous lung cancer, but high TRIB3 protein level did. Such discrepancy indicates that high expression of TRIB3 in squamous lung cancer may be the result of dysregulation of its protein stability as reported[11]. In the future, more experimental evidences should be provided to elucidate how TRIB3 protein is regulated, especially in squamous lung cancer.

PKCα plays complicated roles in cancer, acting either as a tumor promoter or suppressor depending on the tissue origins[12]. In general, PKCα is considered a therapeutic target in cancer

because of its upregulated expression in various cancers, and its strong connections with increases in proliferation, invasion, metastasis, and drug resistance of cancer cells[13]. However, aprinocarsen, an antisense oligonucleotide targeting human PKCα expression, failed in phase III clinical trials evaluating the treatment of various human tumors due to non-achievement of efficacy endpoints and development of associated toxicity[14]. In this study, SAH-JGZ4 moderately decreased PKCα expression, suggesting that PKCα-related toxicity and compensatory activation of other PKC isoforms would be relatively mild. SAH-JGZ4 interfered with the interaction between EGFR and PKCα and suppressed the T654 phosphorylation of EGFR, suggesting that this peptide acts as a competitor for PKCα substrate binding. However, whether this competition is specifically directed at EGFR remains to be explored.

Supplementary References

1. Hu Y, Smyth GK. ELDA: extreme limiting dilution analysis for comparing depleted and enriched populations in stem cell and other assays. *J Immunol Methods* **347**, 70-78 (2009).
2. Grovdal LM, Stang E, Sorkin A, Madshus IH. Direct interaction of Cbl with pTyr 1045 of the EGF receptor (EGFR) is required to sort the EGFR to lysosomes for degradation. *Exp Cell Res* **300**, 388-395 (2004).
3. Aifa S, Johansen K, Nilsson UK, Liedberg B, Lundstrom I, Svensson SP. Interactions between the juxtamembrane domain of the EGFR and calmodulin measured by surface plasmon resonance. *Cell Signal* **14**, 1005-1013 (2002).
4. Lo HW*, et al.* Nuclear interaction of EGFR and STAT3 in the activation of the iNOS/NO pathway. *Cancer Cell* **7**, 575-589 (2005).
5. Du K, Herzig S, Kulkarni RN, Montminy M. TRB3: a tribbles homolog that inhibits Akt/PKB activation by insulin in liver. *Science* **300**, 1574-1577 (2003).
6. Hua F*, et al.* TRB3 interacts with SMAD3 promoting tumor cell migration and invasion. *J Cell Sci* **124**, 3235-3246 (2011).
7. Hua F*, et al.* TRB3 links insulin/IGF to tumour promotion by interacting with p62 and impeding autophagic/proteasomal degradations. *Nat Commun* **6**, 7951 (2015).
8. Li K*, et al.* TRIB3 Promotes APL Progression through Stabilization of the Oncoprotein PML-RARalpha and Inhibition of p53-Mediated Senescence. *Cancer Cell* **31**, 697-710 e697 (2017).

9. Force T, Krause DS, Van Etten RA. Molecular mechanisms of cardiotoxicity of tyrosine kinase inhibition. *Nat Rev Cancer* **7**, 332-344 (2007).
10. Lopez-Bergami P*, et al.* Rewired ERK-JNK signaling pathways in melanoma. *Cancer Cell* **11**, 447-460 (2007).
11. Ding CZ*, et al.* High glucose contributes to the proliferation and migration of non-small cell lung cancer cells via GAS5-TRIB3 axis. *Biosci Rep*, (2018).
12. Konopatskaya O, Poole AW. Protein kinase Calpha: disease regulator and therapeutic target. *Trends Pharmacol Sci* **31**, 8-14 (2010).
13. Isakov N. Protein kinase C (PKC) isoforms in cancer, tumor promotion and tumor suppression. *Semin Cancer Biol* **48**, 36-52 (2018).
14. Paz-Ares L*, et al.* Phase III study of gemcitabine and cisplatin with or without aprinocarsen, a protein kinase C-alpha antisense oligonucleotide, in patients with advanced-stage non-small-cell lung cancer. *J Clin Oncol* **24**, 1428-1434 (2006).
