# Supplementary material for: TRIB3-EGFR interaction promotes lung cancer progression and defines a therapeutic target
Source: Nat Commun. 2020 Jul 21;11:3660. doi: 10.1038/s41467-020-17385-0 (PMC7374170; doi:10.1038/s41467-020-17385-0)
Supplement: Supplementary file 3 — Reporting Summary [file 41467_2020_17385_MOESM3_ESM.pdf]

nature research

Corresponding author(s): Fang Hua and Zhuo-Wei Hu

Last updated by author(s): Jun 16, 2020

Reporting Summary

Nature Research wishes to improve the reproducibility of the work that we publish. This form provides structure for consistency and transparency in reporting. For further information on Nature Research policies, see Authors & Referees and the Editorial Policy Checklist .

Statistics

For all statistical analyses, confirm that the following items are present in the figure legend, table legend, main text, or Methods section.

n/a	Confirmed	
☐	☒	The exact sample size (*n*) for each experimental group/condition, given as a discrete number and unit of measurement
☐	☒	A statement on whether measurements were taken from distinct samples or whether the same sample was measured repeatedly
☐	☒	The statistical test(s) used AND whether they are one- or two-sided<br>*Only common tests should be described solely by name; describe more complex techniques in the Methods section.*
☒	☐	A description of all covariates tested
☒	☐	A description of any assumptions or corrections, such as tests of normality and adjustment for multiple comparisons
☐	☒	A full description of the statistical parameters including central tendency (e.g. means) or other basic estimates (e.g. regression coefficient) AND variation (e.g. standard deviation) or associated estimates of uncertainty (e.g. confidence intervals)
☐	☒	For null hypothesis testing, the test statistic (e.g. *F*, *t*, *r*) with confidence intervals, effect sizes, degrees of freedom and *P* value noted<br>*Give P values as exact values whenever suitable.*
☒	☐	For Bayesian analysis, information on the choice of priors and Markov chain Monte Carlo settings
☒	☐	For hierarchical and complex designs, identification of the appropriate level for tests and full reporting of outcomes
☐	☒	Estimates of effect sizes (e.g. Cohen's *d*, Pearson's *r*), indicating how they were calculated

Our web collection on statistics for biologists contains articles on many of the points above.

Software and code

Policy information about availability of computer code

Data collection	Standard software and the respective analysis tools provided by manufacturers were listed in the methods (PARTEC EPICSXL, Olympus microsystems, Bioer LineGene 9620 PCR, GE BIAcore T200 etc.). No software was used other than that listed in the Methods.
Data analysis	Student's t-test, one-way ANOVA analysis, Pearson's correlation test and Kaplan-Meier analysis were done by using Prism GraphPad 7.0. GSEA analysis was conducted using MSigDB v6.1. Quantitative image analysis was performed with Imaris 9.3.1. The Proteome Microarray data was analyzed using GenePix Pro 6.0. Frequency of tumorigenic cell and probability estimates were computed using Extreme Limiting Dilution Analysis (ELDA) software. The dissociation constant (KD) was calculated according to the BIA-evaluation software. All flow cytometry data were analyzed using FCS Express 6. Immunohistochemistry analysis was performed by Image-Pro Plus 5.1. Western blots images were analyzed by Gel Pro Analyzer 3.2.

For manuscripts utilizing custom algorithms or software that are central to the research but not yet described in published literature, software must be made available to editors/reviewers. We strongly encourage code deposition in a community repository (e.g. GitHub). See the Nature Research guidelines for submitting code & software for further information.

Data

Policy information about availability of data

All manuscripts must include a data availability statement. This statement should provide the following information, where applicable:

- Accession codes, unique identifiers, or web links for publicly available datasets
- A list of figures that have associated raw data
- A description of any restrictions on data availability

All microarray data generated in this study have been deposited at the NCBI Gene Expression Omnibus with the accession code GSE103891 (https://www.ncbi.nlm.nih.gov/geo/query/acc.cgi?acc= GSE103891). Correlation between EGFR and TRIB3 mRNA expression across TCGA lung cancer data sets was analyzed on the following website: http://gepia.cancer-pku.cn. The KM plotter lung cancer dataset was obtained from http://kmplot.com/analysis. All other data supporting the findings of this study are available from the corresponding authors upon reasonable request. A Reporting Summary for this Article is available as a Supplementary Information file. The uncropped gel or blot figures and original data underlying Figures 1-8 and Supplementary Figures 1-9 are provided as a Source

Data file.

Field-specific reporting

Please select the one below that is the best fit for your research. If you are not sure, read the appropriate sections before making your selection.

- [x] Life sciences
- [ ] Behavioural & social sciences
- [ ] Ecological, evolutionary & environmental sciences

For a reference copy of the document with all sections, see nature.com/documents/nr-reporting-summary-flat.pdf

Life sciences study design

All studies must disclose on these points even when the disclosure is negative.

Sample size	For in vitro experiments, at least three biological replicates were achieved for most of the experiments, except for the protein microarray assay. Such sample sizes are typical for the in vitro experiments and sufficient for a statistical analysis. For in vivo experiments, a sample size of n = 6-14 mice was used per experimental group. Sample size was determined based on our previous experience (Nat Commun. 2015;6:7951; Cancer Cell. 2017;31(5):697-710; Gastroenterology. 2019;156(3):708-721), which is sufficient to generate statistically significant results. No statistical method was used to predetermine sample size. For the protein microarray assay, two repeats were set for each protein in the chip.
Data exclusions	No data was excluded in this study.
Replication	Most of the in vitro experiments were repeated independently at least three times except for those specifically indicated in the figure legends. Multiple mice (n = 6-14/group) were used for every in vivo experiment. All the attempts at replication were successful.
Randomization	For animal studies, the mice were earmarked before grouping and then were randomly separated into groups by an independent person; however, no particular method of randomization was used. For other experiments, cells/samples were randomly assigned to groups to avoid bias.
Blinding	Experimenters were blinded to group allocation for IHC staining and grading, quantification of tumor sphere formation. All other experiments were performed in a non-blinded manner, because the experimental design was complicated, the researchers were limited, and blinding feasibility was poor.

Reporting for specific materials, systems and methods

We require information from authors about some types of materials, experimental systems and methods used in many studies. Here, indicate whether each material, system or method listed is relevant to your study. If you are not sure if a list item applies to your research, read the appropriate section before selecting a response.

Materials & experimental systems

n/a	Involved in the study	
[ ]	[x]	Antibodies
[ ]	[x]	Eukaryotic cell lines
[x]	[ ]	Palaeontology
[ ]	[x]	Animals and other organisms
[ ]	[x]	Human research participants
[x]	[ ]	Clinical data

Methods

n/a	Involved in the study	
[x]	[ ]	ChIP-seq
[ ]	[x]	Flow cytometry
[x]	[ ]	MRI-based neuroimaging

Antibodies

Antibodies used	Western Blots: anti-TRIB3 (Abcam, ab75846, 1:1000), anti-TRIB3 (ThermoFisher, PA5-15480, 1:1000), anti-EGFR (CST, #4267, 1:1000), anti-GAPDH (ZSGB-BIO, TA-08, 1:2000), anti-Phospho-p44/42 MAPK (Erk1/2) (Thr202/Tyr204) (CST, #4370, 1:1000), anti-p44/42 MAPK (Erk1/2) (CST, #4695, 1:1000), anti-Phospho-Stat3 (Tyr705) (CST, #9145, 1:1000), anti-Stat3 (CST, #9139, 1:1000), anti-Phospho-Stat5 (Tyr694) (CST, #4322, 1:1000), anti-Stat5 (CST, #94205, 1:1000), anti-phospho-EGF Receptor (Tyr1068) (CST, #3777, 1:1000), anti-PKCα (CST, #2056, 1:1000), anti-phospho-EGFR (Thr654) (Merck, #04-282, 1:500), anti-Myc (MBL, #562, 1:1000), anti-GFP (MBL, #598, 1:1000), anti-HA (MBL, #561, 1:1000), anti-DDK (MBL, PM020), anti-WWP1 (Abcam, ab43791, 1:1000), anti-K63-linkage Specific Polyubiquitin (CST, #5621, 1:1000), anti-K48-linkage Specific Polyubiquitin (CST, #8081, 1:1000), anti-c-Myc (CST, #18583, 1:1000), anti-Oct4 (CST, #2750, 1:1000), anti-KLF4 (CST, #4038, 1:1000), anti-Nanog (CST, #4903, 1:1000), anti-Sox2 (CST, #14962, 1:1000), anti-Epcam (CST, #14962, 1:1000), anti-c-Met (CST, #8198, 1:1000), anti-AXL (CST, #8661, 1:1000), anti-IGF-I Receptor β (CST, #9750, 1:1000), anti-FGFR1 (CST, #9740, 1:1000), anti-Her2 (CST, #4290, 1:1000); Immunofluorescence& Immunohistochemistry: anti-TRIB3 (Abcam, ab137526, 1:100), anti-Rab11(CST, #5589, 1:100), anti-EGFR(Abcam, ab231, 1:100), anti-EGFR (Santa Cruz, R-1, #sc-101 AF488, 1:100), anti-EGFR (CST, #4267, 1:100), anti-EEA1 (Abcam, #ab70521, 1:100), anti-Lamp1 (Abcam, #ab25630, 1:100), anti-PKCα (CST, #2056, 1:100), anti-PKCα (Abcam, # ab32376, 1:100), anti-PKC (Abcam, #ab31, 1:100), Alexa Fluor™ 555 Phalloidin (Invitrogen, #A34055, 1:200); WWP1 (Abcam, ab227213, 1:100); Flow Cytometry: anti-EGFR (Santa Cruz, R-1, sc-101 AF488, 1:100); Elisa: anti-EGFR (Santa Cruz, sc-03, 1:500).

Validation

anti-TRIB3, human, WB and IP, (https://www.abcam.com/trib3-antibody-epr3151y-ab75846.html); anti-TRIB3, human and mouse, WB, (https://www.thermofisher.com/cn/zh/antibody/product/TRIB3-Antibody-Polyclonal/PA5-15480); anti-EGFR, human and mouse, WB, IP, IHC and IF, (https://www.cellsignal.co.uk/products/primary-antibodies/egf-receptor-d38b1-xp-rabbit-mab/4267); anti-GAPDH, human and mouse, WB, (http://www.zsbio.com/product/TA-08); anti-Phospho-p44/42 MAPK (Erk1/2) (Thr202/Tyr204), human and mouse, WB, (https://www.cellsignal.co.uk/products/primary-antibodies/phospho-p44-42-mapk-erk1-2-thr202-tyr204-d13-14-4e-xp-rabbit-mab/4370); anti-p44/42 MAPK (Erk1/2), human and mouse, WB, (https://www.cellsignal.co.uk/products/primary-antibodies/p44-42-mapk-erk1-2-137f5-rabbit-mab/4695); anti-Phospho-Stat3 (Tyr705), human and mouse, WB, (https://www.cellsignal.com/products/primary-antibodies/phospho-stat3-tyr705-d3a7-xp-rabbit-mab/9145); anti-Stat3, human and mouse, WB, (https://www.cellsignal.com/products/primary-antibodies/stat3-124h6-mouse-mab/9139); anti-Phospho-Stat5 (Tyr694), human and mouse, WB, (https://www.cellsignal.com/products/primary-antibodies/phospho-stat5-tyr694-d47e7-xp-rabbit-mab/4322); anti-Stat5, human and mouse, WB, (https://www.cellsignal.com/products/primary-antibodies/stat5-d2o6y-rabbit-mab/94205); anti-phospho-EGF Receptor (Tyr1068), human and mouse, WB, (https://www.cellsignal.com/products/primary-antibodies/phospho-egf-receptor-tyr1068-d7a5-xp-rabbit-mab/3777); anti-PKCα, human and mouse, WB and IF and IP, (https://www.cellsignal.co.uk/products/primary-antibodies/pkca-antibody/2056); anti-phospho-EGFR (Thr(654), human and mouse, WB, (https://www.sigmaaldrich.com/catalog/product/mm/04282?lang=zh&region=CN); anti-Myc, human and mouse, WB and IP, (https://www.mblbio.com/bio/g/dtl/A/?pcd=562); anti-GFP, human and mouse, WB and IP, (https://www.mblbio.com/bio/g/dtl/A/?pcd=598); anti-HA, human and mouse, WB and IP, (https://www.mblbio.com/bio/g/dtl/A/?pcd=561); anti-DDK, human and mouse, WB and IP, (https://ruo.mbl.co.jp/bio/dtl/A/?pcd=PM020); anti-WWP1, human, WB, (https://www.abcam.com/WWP1-antibody-ab43791.html); anti-K63-linkage Specific Polyubiquitin, all, WB, (https://www.cellsignal.com/products/primary-antibodies/k63-linkage-specific-polyubiquitin-d7a11-rabbit-mab/5621); anti-K48-linkage Specific Polyubiquitin, all, WB, (https://www.cellsignal.com/products/primary-antibodies/k48-linkage-specific-polyubiquitin-d9d5-rabbit-mab/8081); anti-c-Myc, human and mouse, WB, (https://www.cellsignal.com/products/primary-antibodies/c-myc-e5q6w-rabbit-mab/18583); anti-Oct4, human, WB, (https://www.cellsignal.com/products/primary-antibodies/oct-4-antibody/2750); anti-KLF4, human and mouse, WB, (https://www.cellsignal.com/products/primary-antibodies/klf4-antibody/4038); anti-Nanog, human, WB, (https://www.cellsignal.com/products/primary-antibodies/nanog-d73g4-xp-rabbit-mab/4903); anti-Sox2, human and mouse, WB, (https://www.cellsignal.com/products/primary-antibodies/sox2-d1c7j-xp-rabbit-mab/14962); anti-Epcam, human, WB, (https://www.cellsignal.com/products/primary-antibodies/epcam-vu1d9-mouse-mab/2929); anti-c-Met, human, WB, (https://www.cellsignal.com/products/primary-antibodies/met-d1c2-xp-rabbit-mab/8198); anti-AXL, human and mouse, WB, (https://www.cellsignal.com/products/primary-antibodies/axl-c89e7-rabbit-mab/8661); anti-IGF-I Receptor β, human and mouse, WB, (https://www.cellsignal.com/products/primary-antibodies/igf-i-receptor-b-d23h3-xp-rabbit-mab/9750); anti-FGFR1, human and mouse, WB, (https://www.cellsignal.com/products/primary-antibodies/fgf-receptor-1-d8e4-xp-rabbit-mab/9740); anti-Her2, human and mouse, WB, (https://www.cellsignal.com/products/primary-antibodies/her2-erbb2-d8f12-xp-rabbit-mab/4290); anti-TRIB3, human, WB, IHC-P and IF, (https://www.abcam.com/TRIB3-antibody-ab137526.html); anti-Rab11, human and mouse, IF, (https://www.cellsignal.com/products/primary-antibodies/rab11-d4f5-xp-rabbit-mab/5589); anti-EGFR, human and mouse, IF, (https://www.abcam.com/egfr-antibody-icr10-ab231.html); anti-EGFR Alexa Fluor 488, human and mouse, IF and FCM, (https://www.scbt.com/p/egfr-antibody-r-1); anti-EEA1, human, IF, (https://www.abcam.com/EEA1-antibody-1G11-Early-Endosome-Marker-ab70521.html); anti-Lamp1, human, IF, (https://www.abcam.com/lamp1-antibody-h4a3-ab25630.html); anti-PKCα, human and mouse, WB and IF (https://www.abcam.com/pkc-alpha-antibody-y124-ab32376.html); anti-PKC, human and mouse, IF, (https://www.abcam.com/pkc-antibody-mc5-ab31.html); Alexa Fluor™ 555 Phalloidin, all, IF, (https://www.thermofisher.com/order/catalog/product/A34055#/A34055); anti-EGFR, human, Elisa, (https://www.scbt.com/p/egfr-antibody-1005).

Eukaryotic cell lines

Policy information about cell lines

Cell line source(s)

NCI-H1703, NCI-H2170, NCI-H157, NCI-H1395, NCI-H1975, A549, NCI-H1650, NCI-H460, BEAS-2B, 293T and HaCaT cells were obtained from The Chinese National Infrastructure of Cell Line Resource, located in Peking Union Medical College (Beijing, China). NCM460 cells were obtained from GuangZhou Jennio Biotech Co.,Ltd. 4T1 Cells were provided by Dr. Bo Huang from Institute of Basic Medicine, Chinese Academy of Medical Sciences & Peking Union Medical College.

Authentication

Cell lines were authenticated by the Chinese National Infrastructure of Cell Line Resource via STR genotyping. We have not authenticated these cell lines by ourselves.

Mycoplasma contamination

Cell lines were routinely tested for potential mycoplasma contamination by using commercial mycoplasma detection kits (Lonza, LT07-418). All tests were negative.

Commonly misidentified lines
(See ICLAC register)

No commonly misidentified cell lines were used in the study.

Animals and other organisms

Policy information about studies involving animals; ARRIVE guidelines recommended for reporting animal research

Laboratory animals

5-6 weeks old male BALB/c nude mice were purchased from HFK Bioscience Co., Ltd (Beijing, China). 5-6 weeks old male NCG/NSG (NOD-Prkdcscidll2rgnull) mice were purchased from Nanjing Biomedical Research Institute of Nanjing University (Nanjing, China). Animals were housed under specific pathogen free conditions with free-water and free-food. No clinical signs were detected. Animals were housed in groups of 4-6 mice per individually ventilated cage in a 12 h light/dark cycle (07:30-19:30 light; 19:30-7:30 dark), with controlled room temperature (23 ± 2°C) and relative humidity (40-50 %).

Wild animals

No wild animals were used in this study.

Field-collected samples	No field-collected samples were used in this study.
Ethics oversight	All experiments using animals were performed in accordance with protocols approved by the Animal Experimentation Ethics Committee of the Chinese Academy of Medical Sciences, and all procedures were conducted in accordance with the guidelines of the Institutional Animal Care and Use Committees of the Chinese Academy of Medical Sciences. All animal procedures were consistent with the ARRIVE guidelines.

Note that full information on the approval of the study protocol must also be provided in the manuscript.

Human research participants

Policy information about studies involving human research participants

Population characteristics	Clinical and genetic characteristics of the patients from whom organoids and PDX were derived are list in Supplementary Table 2 and Supplementary Table 3.
Recruitment	Lung cancer tissues were obtained from Cancer Institute and Hospital, Chinese Academy of Medical Science and the Guangdong provincial people's Hospital.
Ethics oversight	Informed consent was obtained from all participants in accordance with the Declaration of Helsinki. All protocols using human specimens were approved by the Institutional Review Board of the Chinese Academy of Medical Sciences and Peking Union Medical College.

Note that full information on the approval of the study protocol must also be provided in the manuscript.

Flow Cytometry

Plots

Confirm that:

- [x] The axis labels state the marker and fluorochrome used (e.g. CD4-FITC).
- [x] The axis scales are clearly visible. Include numbers along axes only for bottom left plot of group (a 'group' is an analysis of identical markers).
- [x] All plots are contour plots with outliers or pseudocolor plots.
- [x] A numerical value for number of cells or percentage (with statistics) is provided.

Methodology

Sample preparation	About 1*10e6 single cells were suspended in PBS and incubated with Alexa-Fluro 488 anti-EGFR antibody at room temperature for 20 min.
Instrument	PARTEC EPICSXL
Software	FCS Express 6
Cell population abundance	No sorting was performed.
Gating strategy	Gating strategy is provided in the Supplementary Fig.2b.

- [x] Tick this box to confirm that a figure exemplifying the gating strategy is provided in the Supplementary Information.
